# Initial surgical management of injuries to the lower extremities in patients with multiple and/or severe injuries – A systematic review and clinical practice guideline update

**DOI:** 10.1007/s00068-024-02662-0

**Published:** 2024-11-05

**Authors:** Kai Oliver Jensen, Barbara Prediger, Nadja Könsgen, Michel Paul Johan Teuben

**Affiliations:** 1 Department of Traumatology, Zurich University Hospital, Zurich, Switzerland; 2 Institute for Research in Operative Medicine (IFOM), Witten/Herdecke University, Cologne, Germany

**Keywords:** Initial surgical management, Lower extremities, Fractures, Dislocation, Compartment syndrome, Polytrauma guideline

## Abstract

**Purpose:**

Our aim was to develop new evidence-based and consensus-based recommendations for the initial inhospital management of lower-extremity injuries in patients with multiple and/or severe trauma. This guideline topic is part of the 2022 update of the German Guideline on the Treatment of Patients with multiple and/or severe Injuries.

**Methods:**

MEDLINE and Embase were systematically searched to May 2021. Randomised controlled trials, prospective cohort studies, and comparative registry studies were included if they compared interventions for the initial surgical and non-surgical management of fractures, dislocations or vascular injuries of the lower extremities in patients with multiple and/or severe trauma. We considered patient-relevant clinical outcomes such as mortality, complication rates, length of stay, and function. Risk of bias was assessed using NICE 2012 checklists. The evidence was synthesised narratively, and expert consensus was used to develop recommendations and determine their strength.

**Results:**

Eleven studies were identified. They addressed time to definitive fixation (*n* = 10 studies) and amputation (*n* = 1). Two new recommendations were developed, one was modified. All recommendations achieved strong consensus.

**Conclusion:**

This systematic literature review and subsequent expert consensus process resulted in the following new key recommendations. It is recommended that isolated and multiple lower-extremity fractures are managed with primary definitive fixation in patients whose condition is stable. Patients condition is not considered stable should be managed with primary temporary fixation. In addition, it is recommended that dislocations of the lower extremities are reduced and immobilised as early as possible.

**Supplementary Information:**

The online version contains supplementary material available at 10.1007/s00068-024-02662-0.

## Introduction

The treatment of polytrauma patients has changed massively over the years and the implementation of novel treatment guidelines has resulted in improved short-term patient outcomes (e.g. survival, complications, ICU and hospital lengths of stay) as well as long-term patient outcomes (e.g. return to work and functional results) [1, 2]. Protocols for the treatment of patients with multiple trauma vary between countries and institutions. The German Polytrauma Guideline was last updated in 2016 [3]. Since the 2016 guideline version was published, a number of novel studies on the surgical management of lower-extremity injuries have become available. For this reason, the aim of this study was to review the current literature and to update the evidence-based and consensus-based recommendations on the surgical management of lower-extremity injuries in patients with multiple and/or severe trauma. This guideline topic is part of the 2022 update of the German Guideline on the Treatment of Patients with Multiple and/or Severe Injuries [4]. Consensus recommendations for key clinical issues were sought. The following topics have been addressed: treatment strategies for isolated or combined lower-extremity fractures, the definitive treatment of femur fractures and dislocations, the use of antibiotics, and amputation versus limb-salvage criteria.

## Methods

This guideline topic is part of the 2022 update of the German Guideline on the Treatment of Patients with Multiple and/or Severe Injuries [4]. The guideline update is reported according to the RIGHT tool [5], the systematic review part according to the Preferred Reporting Items for Systematic Reviews and Meta-Analyses (PRISMA) 2020 reporting guideline [6]. The development and updating of recommendations followed the standard methodology set out in the guideline development handbook issued by the German Association of the Scientific Medical Societies (AWMF) [7]. All methods were defined a priori, following the methods report of the previous guideline version from July 2016 [1] with minor modifications, as detailed below. The Discussion section of this publication is a direct translation of the original guideline text [4].

### PICO questions and eligibility criteria

The overarching Population, intervention, comparison, and outcome (PICO) question for this topic area was:

*In adult patients (≥ 14 years) with known or suspected polytrauma and/or severe injuries*,* does a specific initial surgical approach to the management of lower-extremity injuries improve patient-relevant outcomes compared to any other intervention?*

Main PICO questions were retained from the previous guideline version. In addition, the participating professional societies involved in guideline development were asked to submit new PICO questions.

The full set of predefined PICO questions is listed in Table S1 (Online Resource 1). The study selection criteria in the PICO format are shown in Table 1.

**Table 1:** Predefined selection criteria

Population	adult patients (≥ 14 years) with polytrauma and/or severe injuries^a^
Intervention /comparison	(surgical) treatment of fractures, dislocations and vascular injuries of the lower extremities (excluding the pelvis)
Outcomes	any patient-relevant outcome, such as mortality, length of stay, function
Study type	• comparative, prospective studies (randomised controlled trials, cohort studies) • comparative registry^c^ data (incl. case-control studies) • systematic reviews based on the above primary study types
Language	English or German
Other inclusion criteria	• full text of study published and accessible • study matches predefined PICO question
Exclusion criteria	• multiple publications of the same study without additional information

^a^ Defined by an Injury Severity Score (ISS) > 15, Glasgow Coma Scale (GCS) < 9, or comparable values on other scales, or, in the prehospital setting, clinical suspicion of polytrauma/severe injury with a need for life-saving interventions

^c^ Using the Agency for Healthcare Research and Quality (AHRQ) definition of registries [8]

### Literature search

An information specialist systematically searched for literature in MEDLINE (Ovid) and Embase (Elsevier). The search strategy described in the 2011 Guideline was used with modifications. It contained index (MeSH/Emtree) and free text terms for the population and intervention. The searches were completed on 6 May 2021. The start date for update searches was 1 January 2009. Table S2 (Online Resource 1) provides details for all searches. Clinical experts were asked to submit additional relevant references.

### Study selection

Study selection was performed by two reviewers in a two-step process using the predefined eligibility criteria: (1) title/abstract screening of all references retrieved from database searches using Rayyan software [9] and (2) full-text screening of all articles deemed potentially relevant by at least one reviewer at the title/abstract level in Endnote (Endnote, Version: 20 [Software]. Clarivate, Boston, Massachusetts, USA. https://endnote.com/). Disagreements were resolved through consensus or by consulting a third reviewer. The reasons for full-text exclusion were recorded (Table S3, Online Resource 1).

### Assessment of risk of bias and level of evidence

Two reviewers sequentially assessed the risk of bias of included studies at study level using the relevant checklists from the NICE guidelines manual 2012 [10] and assigned each study an initial level of evidence (LoE) using the Oxford Centre for Evidence-based Medicine Levels of Evidence (2009) [11]. Any disagreements were resolved through consensus or by consulting a third reviewer.

### Data extraction and data items

Data were extracted into a standardised data table by one reviewer and checked by another. A predefined data set was collected for each study, consisting of study characteristics (study type, aims, setting), patient selection criteria and baseline characteristics (age, gender, injury scores, other relevant variables), intervention and control group treatments (including important co-interventions), patient flow (number of patients included and analysed), matching/adjusting variables, and data on outcomes for any time point reported.

### Outcome measures

Outcomes were extracted as reported in the study publications. For prospective cohort studies and registry data, preference was given to data obtained after propensity-score matching or statistical adjustment for risk-modulating variables over unadjusted data.

### Synthesis of studies

Studies were grouped by interventions. An interdisciplinary expert group used their clinical experience to synthesise studies narratively by balancing beneficial and adverse effects extracted from the available evidence. Priority was given to reducing mortality, immediate complications, and long-term adverse effects. Clinical heterogeneity was explored by comparing inclusion criteria and patient characteristics at baseline as well as clinical differences in the interventions and co-interventions.

### Development and updating of recommendations

For each PICO question, the following updating options were available: (1) the recommendation of the preceding version remains valid and requires no changes (“confirmed”); (2) the recommendation requires modification (“modified”); (3) the recommendation is no longer valid or required and is deleted; (4) a new recommendation needs to be developed (“new”). An interdisciplinary expert group of clinicians with expertise in trauma, orthopaedic trauma, and acute care reviewed the body of evidence, drafted recommendations based on the homogeneity of clinical characteristics and outcomes, the balance between benefits and harms as well as their clinical expertise, and proposed grades of recommendation (Table 2). In the absence of eligible evidence, good practice recommendations were made based on clinical experience, data from studies with a lower level of evidence, and expert consensus in cases where the Guideline Group felt a statement was required due to the importance of the topic. These were not graded, and instead labelled as good (clinical) practice points (GPP). For GPPs, the strength of a recommendation is presented in the wording shown in Table 2.

**Table 2:** Grading of recommendations

Symbol	Grade of recommendation	Description	Wording (examples)
⇑⇑	A	strong recommendation	“use…”, “do not use…”
⇑	B	recommendation	“should use…”, “should not use…”
⇔	0	open recommendation	“consider using…”, “… can be considered”

### Consensus process

The Guideline Group finalised the recommendations during web-based, structured consensus conferences on 6 May 2021 and 26 January 2022 via Zoom (Zoom, Version: 5.x [Software]. Zoom Video Communications, Inc., San José, California, USA. https://zoom.us). A neutral moderator facilitated the consensus conference. Voting members of the Guideline Group were delegates of all participating professional organisations, including clinicians, emergency medical services personnel and nurses, while guideline methodologists attended in a supporting role. Members with a moderate, thematically relevant conflict of interest abstained from voting on recommendations, members with a high, relevant conflict of interest were not permitted to vote or participate in the discussion. Attempts to recruit patient representatives were unsuccessful. A member of the expert group presented recommendations. Following discussion, the Guideline Group refined the wording of the recommendations and modified the grade of recommendation as needed. Agreement with both the wording and the grade of recommendation was assessed by anonymous online voting using the survey function of Zoom. Abstentions were subtracted from the denominator of the agreement rate. If no consensus was reached, the statement was returned to the working group for rewriting. Consensus strength was classified as shown in Table 3.

**Table 3:** Classification of consensus strength

Description	Agreement rate
strong consensus	> 95% of participants
consensus	> 75 to 95% of participants
majority approval	> 50 to 75% of participants
no approval	< 50% of participants

Recommendations were accepted if they reached consensus or strong consensus. For consensus recommendations with ≤ 95% agreement, diverging views by members of the Guideline Group were detailed in the background texts. Recommendations with majority approval were returned to the expert group for revision and further discussion at a subsequent consensus conference. Recommendations without approval were considered rejected.

### External review

During a four-week consultation phase, the recommendations and background texts were submitted to all participating professional organisations for review. Comments were collected using a structured review form. The results were then assessed, discussed and incorporated into the text by the guideline coordinator with the relevant author group.

The guideline was adopted by the executive board of the German Trauma Society on 17 January 2023.

### Quality assurance

The guideline recommendations were reviewed for consistency between guideline topic areas by the steering group. Where necessary, changes were made in collaboration with the clinical leads for all topic areas concerned. The final guideline document was checked for errors by the guideline chair and methodologist.

## Results

**Fig. 1:**
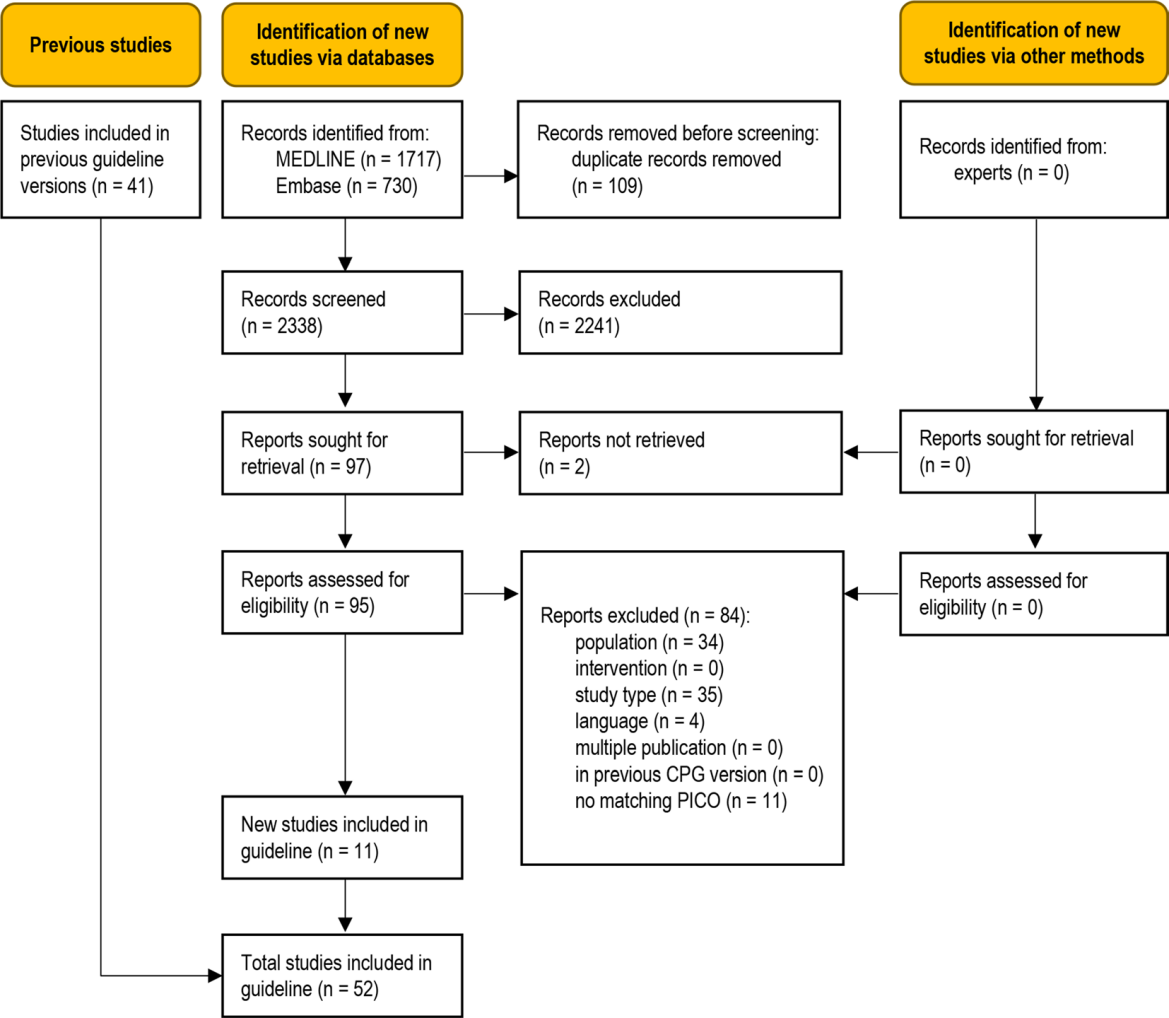
Modified PRISMA 2020 flow diagram showing the systematic literature search and selection of studies

The database searches identified 2338 unique records (Fig. 1). No additional records were obtained from clinical experts. Eleven studies were eligible for this update [12–22]. A total of 84 full-text articles were excluded (Table S3, Online Resource 1).

### Characteristics of studies included in this update

Study characteristics, main outcomes, levels of evidence, and risk-of-bias assessments are presented in Table 4. Full details are provided in Table S4, Online Resource 1. The evidence included one RCT [20], one prospective cohort study [22], and nine comparative registry studies [12–19, 21]. Four studies were performed in North America, four in Europe, and one was performed in military settings in Afghanistan and Iraq. The setting was not reported in two studies. Eligible patient populations were adults with severe injuries and known injuries to the lower extremities. All studies, with the exception of one, were limited to femoral fractures [12–14, 16–22].

**Table 4:** Characteristics of studies included in the update (see table S4, Online Resource 1 for details)

Study, ref, design	Population	Interventions (*N* patients)	Main outcomes (selection)*	LoE, risk of bias (RoB)^§^, comments
Primary management of fractures				
Blair 2019 [12] Comparative registry study	Patients with closed and open femoral shaft fractures^a^	*N* = 10,072 IG1: DF < 24 h (*N* = 6569) IG2: DF 24–48 h (*N* = 1327) IG3: DF 48–72 h (*N* = 631) CG: DF < 72 h (*N* = 1545)	**Univariate analysis** *Inhospital mortality OR (95% CI)* ≥ 24 h vs. reference < 24 h: 1.001 (1.000-1.002), *p* = 0.028 24–48 h vs. reference < 24 h: n.s. 48–72 h vs. reference < 24 h: n.s. > 72 h vs. reference < 24 h: n.s.	LoE: 2b High risk of selection bias
Bläsius 2021 [13] Comparative registry study	Patients with femur fractures^b^	*N* = 13,091 IG1: TC (cast/brace or no immobilisation) (*N* = 1601) IG2: EF (part of DCO) (*N* = 5249) CG: ETC (*N* = 6241)	**RISC-II-adjusted standardisation** *Mortality*,* SMR (95% CI)* IG1: 1.30 (1.19–1.41) vs. IG2: 0.85 (0.75–0.95) vs. CG: 0.83 (0.77–0.90)	LoE: 2b High risk of selection bias
Cantu 2014 [14] Comparative registry study	Patients with unilateral closed or open femoral shaft fractures^a^	*N* = 2323 IG: <12 h IMN from hospital presentation (N = n.r.) IG: 12–24 h IMN from hospital presentation (N = n.r.) IG: 24–48 h IMN from hospital presentation (N = n.r.) CG: >48 h to 30 days IMN from hospital presentation (N = n.r.)	**Individual and multiple predictor models** *Inhospital mortality rates*,* ISS 16–25*,* % (95% CI)* < 12 h: 1.53 (0.84–2.77) 12–24 h: 2.65 (1.30–5.41) 24–48 h: 1.76 (0.59–5.28) 48 h-30d: 2.68 (1.05–6.84) *Inhospital mortality rates*,* ISS > 25*,* % (95% CI)* < 12 h: 5.24 (3.59–7.66) 12–24 h: 1.36 (0.36–5.14) 24–48 h: 1.26 (0.33–4.83) 48 h-30d: 5.90 (3.63–9.60)	LoE: 2b High risk of selection bias
Flagstad 2021 [16] Comparative registry study	Patients with bilateral femur fractures	*N* = 246 IG: IMN during two separate (two-stage) procedures (*N* = 58) CG: IMN during a single (single-stage) procedure (*N* = 188)	*Inhospital mortality*,* n (%)* IG 0 (0.0) vs. CG: 5 (2.7), *p* = 0.22	LoE: 2b High risk of selection bias
Morshed 2009 [17] Comparative registry study	Patients with closed or open femoral shaft fractures^a^	*N* = 3069 IG: DF < 12 h (*N* = 1759) IG: DF 12–24 h (*N* = 540) IG: DF 24–48 h (*N* = 359) IG: DF 48–120 h (*N* = 272) CG: DF > 120 h (*N* = 139)	**Standardised risk ratio analysis** *Inhospital mortality (referent group < 12 h)*,* RR (95% CI)* 12 to 24 h: 0.47 (0.14 to 1.11); *p* = 0.07 24 to 48 h 0.94 (0.44 to 1.76), *p* = 0.85 48 to 120 h: 0.58 (0.21 to 1.09); *p* = 0.09 > 120 h: 0.43 (0.09 to 0.94), *p* = 0.05	LoE: 2b High risk of selection bias
Morshed 2015 [18] Comparative registry study	Patients with closed or open femoral shaft fractures^c^	*N* = 2949 IG: DF < 12 h (*N* = 1685) IG: DF 12–24 h (*N* = 518) IG: DF 24–48 h (*N* = 347) IG: DF 48–120 h (*N* = 263) CG: DF > 120 h (*N* = 136)	**Median regression**,** deaths excluded** *Length of stay (referent group < 12 h)*,* median difference in days (95% CI)* 12 to 24 h: -0.64 (-1.53, 0.3.8), *p* = 0.2949 24 to 48 h: 0.24 (-0.96, 1.61), *p* = 0.6346 48 to 120 h: 2.53 (0.27, 4.13), *p* = 0.0167 > 120 h: 0.55 (-2.04, 1.61), *p* = 0.7674	LoE: 2b High risk of selection bias
Richards 2020 [19] Comparative registry study	Patients with femur fractures^b^	*N* = 279 IG: early fixation (*N* = 160) CG: late fixation (*N* = 119)	**Cox proportional hazards model** *Multiple organ failure within 28 days of injury*,* % (95% CI)* HR = 3.21, 95% CI, 1.48-7.00; *p* < 0.01	LoE: 2b High risk of selection bias
Rixen 2016 RCT[20]	Patients with femoral shaft fractures^d^	*N* = 30 IG: EF and secondary reamed IMN (*N* = 16) CG: primary reamed nailing (*N* = 14)	*Maximum SOFA score within 28 days after trauma*,* mean ± SD* IG: 8.7 ± 3.8 vs. CG: 9.6 ± 5.1, *p* = 0.510	LoE: 1b Unclear RoB
Steinhausen 2014 [21] Comparative registry study	Patients with bilateral femoral shaft fractures	*N* = 379 IG1: DCO with bilateral EF (*N* = 193) IG2: ETC with bilateral primary definitive fixation *N* = 95) IG3: ETC of one femoral shaft fracture and DCO of the other fracture (mixed) (*N* = 59) CG: no fixation (*N* = 32)	*Inhospital mortality*,* n (%)* IG1: 26 (13.5) vs. IG2: 8 (8.4) vs. IG3: 1 (1.7) vs. CG: 21 (65.6)	LoE: 2b High risk of selection bias
Stojiljković 2009 [22] Prospective cohort study	Patients with closed femoral fractures	*N* = 70 IG1: external fixation (*N* = 9) IG2: conversion from external fixation to internal fixation (*N* = 5) CG: internal fixation and use of the Mitković self-dynamisable internal fixator (*N* = 56)	*Healing time in months*,* median* IG1: (SD) 6.11 (0.81) vs. CG: n.r., but significantly longer (*p* < 0.005) *Bad functional outcome: n (%)* IG1: 1 (11.1) vs. IG2: 0 (0) vs. CG: 3 (5.4) *Poor functional outcome: n (%)* IG1: 1 (11.1) vs. IG2: 0 (0) vs. CG: 0 (0) *Good functional outcome: n (%)* IG1: 5 (55.6) vs. IG2: 2 (40) vs. CG: 15 (26.8) *Excellent functional outcome: n (%)* IG1: 2 (22.2) vs. IG2: 3 (60) vs. CG: 38 (67.9)	LoE: 2b High risk of selection bias
Amputation				
Doukas 2013 [15] Comparative registry study	Patients with major lower-extremity injuries sustained in the military setting	*N* = 324 *Unilateral lower-limb injury* IG: Amputation (*N* = 113) CG: Salvage (*N* = 126) *Bilateral lower-limb injury* IG1: Bilateral amputation (*N* = 39) IG2: Amputation and salvage (*N* = 30) CG: Bilateral salvage (*N* = 16)	**Multivariate regression** *Unilateral lower-limb injury* *SMFA scores*,* mean* Total dysfunction: IG 21.5 vs. CG: 29.8, *p* < 0.01 Mobility: IG 27.5 vs. CG: 37.2, *p* < 0.01 Daily activities: IG 20.4 vs. CG: 27.9, *p* < 0.05 Emotional status: IG 37.6 vs. CG: 47.9, *p* < 0.01 Arm/hand function: IG 2.1 vs. CG: 8.2, *p* < 0.01 *Bilateral lower-limb injury* *SMFA scores*,* mean* Total dysfunction: IG1: 22.2 vs. IG2: 24.0‡ vs. CG: 30.0 Mobility: IG1: 30.2 vs. IG2: 30.8‡ vs. CG: 40.3 Daily activities: IG1: 22.8, IG2: 27.5, CG: 26.8 Emotional status: IG1: 33.2‡ vs. IG2: 32.0† vs. CG: 44.4 Arm/hand function: IG1: 3.0‡ vs. IG2: 5.1 vs. CG: 10.0 †Significantly different (*p* < 0.01) from patients with unilateral salvage ‡ Significantly different (*p* < 0.05) from patients with unilateral salvage	LoE: 2b High risk of selection and attrition bias

^*^ Data for IG versus CG unless otherwise specified; for OR, the CG is the reference group unless otherwise specified. ^§^ Risk of bias: low RoB = RoB low for all domains; unclear RoB = RoB unclear for at least one domain, no high RoB in any domain; for studies with high RoB, all domains with high RoB are named, with RoB low or unclear for all other domains (for full details Table S4, Online Resource 1). ^a^ ISS > 15, ^b^ AIS ≥ 3, ISS ≥ 9. ^c^ ISS ≥ 15; ^d^ ISS ≥ 16. Abbreviations: adj., adjusted; CG, control group; d, days; DCO, damage control orthopaedics; DF, definitive fixation; ED, emergency department; EF, temporary external fixation; ETC, early total care; h, hours; HR, hazard ratio; IG, intervention group; IMN, intramedullary nailing; IQR, interquartile range; ISS, injury severity score; ITT, intention-to-treat; MD, mean difference; NISS, New Injury Severity Score; n.r., not reported; n.s., not significant; OR, odds ratio; PH, prehospital; RBC, red blood cell; PBO, placebo; RCT, randomised controlled trial; RR, risk ratio; SBP, systolic blood pressure; SD, standard deviation; SMR, standardised mortality rate; SMFA, Short Musculoskeletal Function Assessment; SOFA, sepsis-related organ failure assessment; TC, conservative treatment; unadj., unadjusted; y, years

### Risk-of-bias assessment for included studies and levels of evidence

No study was judged to be of low risk of bias in any domain. The risk of selection bias was high in ten studies. The risk of performance bias was unclear in all studies and the risk of detection bias was unclear in eight studies due to insufficient reporting.

### Recommendations

Compared with the previous version of the German Polytrauma Guideline, one recommendation was modified, and two additional recommendations were developed based on the updated evidence (Table 5). All achieved strong consensus. Ten recommendations from the 2016 Guideline were not retained in the 2022 update (Table S5, Online Resource 1).

**Table 5:** List of recommendations with grade of recommendation and strength of consensus

No.	GoR	Evidence, consensus^a^	Recommendation	Status 2022	
*Key recommendation*					
1	B ⇑	[12, 14, 16–22] 100%	If possible, isolated and multiple lower-extremity fractures should be managed with primary definitive fixation in patients whose condition is stable.	New	
2	B ⇑	[12, 14, 16–22] 100%	If possible, patients whose condition is not considered stable should be managed with primary temporary fixation.	New	
*Femur fractures*					
3	B ⇑	[23–37] 100%	Locked intramedullary nailing should be the surgical procedure of choice for the definitive treatment of femoral shaft fractures in polytrauma patients.	Confirmed	
*Dislocations*					
4	A ⇑⇑	[38–40] 100%	Reduce and immobilise dislocations of the lower extremities as early as possible.	Modified	
*Antibiotic prophylaxis*					
5	A ⇑⇑	[41–45] 100%	Administer perioperative prophylactic antibiotics in the surgical management of both closed and open lower-extremity fractures.	Confirmed	
*Vascular injuries*					
6	B ⇑	– 100%	If possible, depending on overall injury severity, the surgical or endovascular management of vascular injuries to the lower extremities should be performed as soon as possible, i.e. immediately after the treatment of life-threatening injuries.	Confirmed	
*Compartment syndrome*					
7	A ⇑⇑	[46–49] 100%	Immediately treat compartment syndrome of the lower extremity with compartment decompression and fixation of a concomitant fracture.	Confirmed	
*Amputation*					
8	B ⇑	[50–58] 100%	The decision to salvage or amputate a limb following a severe lower-extremity injury should be individualised to the patient. The local and general condition of the patient plays a key role in this decision.	Confirmed	

GoR, grade of recommendation

## Discussion

### Rationale for recommendations

(1) It is recommended that isolated and multiple lower-extremity fractures are managed with primary definitive fixation in patients whose condition is stable. (2) Inpatients whose condition is not considered stable primary temporary fixation is recommended.

There are two completely different approaches to the management of isolated lower-extremity long-bone fractures: (a) primary definitive fixation and (b) a two-stage procedure with secondary definitive fixation. These two treatment approaches or a combination of treatment approaches can also be used in the management of combined lower-extremity long-bone injuries. In the following, the long bones of the lower extremities are addressed separately from proximal to distal. First, the focus is on the management of isolated injuries, i.e. proximal femoral fractures, femoral shaft fractures, distal femoral fractures, proximal tibial fractures, tibial shaft fractures, distal tibial fractures, and ankle fractures. Then treatment options for bilateral fractures and combined injuries are evaluated.

### Isolated proximal femoral fractures

There are no controlled studies that address the management of **isolated proximal femoral fractures** specifically in multiply injured patients. The studies referred to below investigated patients with isolated proximal femoral fractures as well as polytraumatised patients with a proximal femoral fracture [1–3]. Depending on their localisation, proximal femoral fractures are divided into intracapsular, extracapsular (trochanteric), and subtrochanteric fractures.

Intracapsular femoral head fractures (Pipkin fractures) are rare and often associated with hip dislocation and/or acetabular fractures. Surgical treatment options include the removal of small osteochondral fragments, refixation, and femoral head reconstruction. Whereas femoral neck fractures are common in elderly people after minor trauma, they are usually the result of high-energy trauma in young people and are then often associated with multiple other injuries. Fixation with (cannulated) screws or similar procedures that preserve the femoral head are the preferred treatment in young patients [4–9]. Prosthetic replacement is reported to be equally effective in elderly patients [6–13]. Bhandari et al. [14] and Parker et al. [15–17] conducted meta-analyses and found that internal fixation of isolated femoral neck fractures led to a substantially higher rate of revision surgery, whereas arthroplasty was associated with higher infection rates, greater blood loss and longer operating time and possibly resulted in an increase in mortality rates [14]. An advantage of a bipolar prosthesis over total hip arthroplasty in the management of polytrauma patients has so far not been identified [15–19].

Extracapsular fractures can be definitively managed with an extramedullary sliding hip screw that is attached to a plate (dynamic hip screw, Medoff plate, etc.) or an intramedullary implant (proximal femoral nail, Gamma nail, etc.) [7–9, 15–17, 20–36]. Definitive surgery at the appropriate time is generally considered the standard treatment for proximal femoral fractures [14, 16, 17, 20, 37–43].

There is no evidence from randomised studies on the timing of fracture management, and observational studies reached varying conclusions [42, 44–47]. Early surgery (within 24–36 h) after physiological stabilisation is recommended for the majority of patients. Unnecessary delays in surgery can increase complication rates (decubitus ulcers, pneumonia). Indications for emergency surgery are open fractures, fractures with vascular and/or nerve injuries, and fractures with compartment syndrome. If surgery must be significantly delayed (> 48 h), joint-spanning external fixation can be used temporarily (or permanently, if necessary). Possible complications are bleeding, infection, wound healing problems, avascular femoral head necrosis, non-union, rotational deformity, limited range of motion, prosthetic dislocation, thrombosis, and embolism [48].

### Isolated femoral shaft fractures

Only a minority of all available controlled studies on **isolated femoral shaft fractures** in polytrauma patients used a prospective or randomised study design [49–68]. The majority of studies are based on retrospective data. Surgical stabilisation (definitive fixation with an intramedullary nail or a plate, or two-stage fixation with primary external fixation and secondary definitive fixation with an intramedullary nail or a plate) is considered the standard treatment for femoral shaft fractures. A randomised controlled study did not provide significant results regarding the timing and strategy of femoral fracture management in severely injured patients because study was underpowered [69].

Early definitive management is considered safe in stable polytraumatised patients. In addition, this approach is associated with decreased rates of mortality and systemic complications [70]. Delayed definitive fracture management on the basis of damage control principles is the preferred treatment for unstable patients [71–74]. Definitive fracture management beyond 48 h is, however, associated with increased mortality in severely injured patients [71, 75]. Similar results and recommendations were reported for bilateral femoral shaft fractures [76, 77]. For details on the recommended treatment of femoral shaft fractures, see recommendation 3.

Indications for emergency surgery are open fractures, fractures with vascular and/or nerve injuries, and fractures with compartment syndrome. Early definitive fixation is the preferred treatment for haemodynamically stable patients. Most authors consider intramedullary nailing to be the gold standard [49–52].

### Isolated distal femoral fractures

There are no controlled studies that address the management of **isolated distal femoral fractures** specifically in multiply injured patients. The studies referred to below investigated non-polytraumatised patients with isolated distal femoral fractures as well as polytraumatised patients with distal femoral fractures. Surgery is considered the standard treatment for distal femoral fractures. Indications for emergency surgery are open fractures, fractures with vascular and/or nerve injuries, and fractures with compartment syndrome. Early definitive fixation is the preferred treatment for haemodynamically stable patients. Depending on the type of fracture, both intra-articular fractures and non-intra-articular fractures of the distal femur can be managed with open or closed reduction and fixation with a plate (less invasive stabilisation system [LISS], angled plate, etc.) or a retrograde nail [78–84]. Joint-spanning external fixation can be used temporarily in haemodynamically unstable patients or in a damage control situation.

Possible complications are bleeding, infection, wound healing problems, non-union, rotational deformity, limited range of motion, thrombosis, embolism, and early osteoarthritis.

Primary definitive fixation is considered to be contraindicated in haemodynamically stable patients with unstable open distal grade III femoral fractures. In these cases, other procedures such as external fixation can be used to stabilise a fracture [85].

### Isolated proximal tibial fractures

There are no controlled studies that address the management of **isolated proximal tibial fractures** specifically in multiply injured patients. The studies referred to below investigated patients with isolated proximal tibial fractures as well as polytraumatised patients with proximal tibial fractures.

These fractures can be primarily treated by immobilisation with a splint. Non-displaced fractures are managed nonoperatively with non-weight-bearing and functional therapy. If required, surgical fixation may be performed in order to prevent secondary displacement. Surgical management is considered the standard treatment for displaced proximal tibial fractures [86, 87]. Depending on fracture complexity and articular surface involvement, a variety of procedures can be used, e.g. plate systems (conventional, fixed-angle less invasive stabilisation system [LISS], etc.), tibial nails, screws, and fixation systems [88–91]. Internal fixation must allow for articular surface reconstruction, maintain fracture reduction, ensure stability during exercises, and minimise perioperative soft-tissue damage. Minor displacement can also be managed with arthroscopically assisted and radiologically controlled reduction and percutaneous screw fixation [92]. Indications for emergency surgery are open fractures, fractures with vascular and/or nerve injuries, and fractures with compartment syndrome. In these cases, an external fixator may be applied until the condition of the soft tissues allows definitive treatment to be performed. In haemodynamically stable patients, the preferred treatment is early elective definitive fixation after initial swelling has subsided (e.g. after three to five days). Tibial plateau fractures are associated with injuries to the menisci in up to 50% and with injuries to the ligaments in up to 25% of patients [93].

Possible complications [94] are bleeding, infection, wound healing problems, non-union, rotational deformity, limited range of motion, thrombosis, embolism, and early osteoarthritis.

### Isolated tibial shaft fractures

There are no controlled studies that address the optimum management of **isolated tibial shaft fractures** specifically in multiply injured patients. A core requirement is that treatment be tailored to the patient’s overall condition. As a result of the limited soft-tissue envelope around the distal half of the tibia, the treatment strategy is often guided by the condition of the soft tissue and not by the fracture itself.

Stable fractures with minimum displacement can be managed nonoperatively by plaster cast immobilisation [95]. Surgical management is considered the standard treatment for unstable tibial shaft fractures and usually involves intramedullary nailing [60, 96, 97]. Indications for emergency surgery are open fractures, fractures with vascular injuries, and fractures with compartment syndrome. Early definitive fixation is the preferred treatment for haemodynamically stable patients. If surgery must be significantly delayed (> 48 h) or if an extensive open injury with a high degree of contamination is present, an external fixator can be applied temporarily (or permanently, if necessary) [98].

Bhandari et al. [99] investigated open tibial shaft fractures in a meta-analysis. They found that the use of unreamed intramedullary nails, compared with external fixators, reduced the risk of reoperation, malunion, and superficial infection. A comparison of reamed and unreamed nails showed a reduced risk of reoperation with the reamed technique. In a prospective randomised study, reamed nailing in patients with closed fractures was also associated with lower rates of secondary operations and malunion compared with unreamed nailing [100]. Tibial shaft fractures are associated with ligamentous injuries in up to 22% of cases. Possible complications are bleeding, infection, wound healing problems, soft-tissue necrosis requiring plastic surgery (flaps), non-union, rotational deformity, limited range of motion, thrombosis, and embolism.

### Isolated distal tibial fractures

There are no controlled studies that address the isolated management of **isolated distal tibial fractures** specifically in multiply injured patients. The studies referred to below investigated patients with isolated distal tibial fractures as well as polytraumatised patients with distal tibial fractures.

Surgery is considered the standard treatment for distal tibial fractures. As a result of the limited soft-tissue envelope around the distal tibia (and the pilon), the treatment strategy is often guided by the condition of the soft tissue and not by the fracture itself. Indications for emergency surgery are open fractures, fractures with vascular injuries, and fractures with compartment syndrome. Early definitive fixation is the preferred treatment for haemodynamically stable patients. Fractures of the distal tibia without involvement of the pilon can be managed with intramedullary nailing. Fixed-angle plate fixation is an alternative to intermedullary nailing, especially when the plate is inserted through a small incision. In patients with an additional fracture of the distal fibula, additional plate fixation of the fibula is recommended (in order to provide a more stable construct and avoid distal deviation of the axis) [101–109]. Open reduction and internal fixation is the standard treatment for fractures involving the pilon [110–113]. If surgery must be significantly delayed (> 48 h), for example because of severe swelling or open contamination, a joint-spanning external fixator can be applied temporarily (or permanently, if necessary) with or without percutaneous fixation of the articular surface (with screws, K-wires). Possible complications are bleeding, infection, wound healing problems, soft-tissue necrosis requiring plastic surgery (flaps), non-union, rotational deformity, limited range of motion, thrombosis, embolism, and early osteoarthritis.

### Isolated ankle fractures

There are no controlled studies that address the isolated management of **isolated ankle fractures** specifically in multiply injured patients. The studies referred to below investigated patients with isolated ankle fractures as well as polytraumatised patients with ankle fractures.

Surgical management and the type of fibular fracture fixation depend, for example, on the overall pattern of injury in polytraumatised patients. Some authors prefer external fixation for patients with an ISS > 25 or 29 and/or an AIS > 3 thoracic injury [95, 114, 115]. Moreover, the type of material that is used for fixation depends on the type of fracture.

Proximal fibula: In patients with Maisonneuve fractures, the distal fibula should be surgically fixated to the tibia at the level of the syndesmosis [116] using two tricortical syndesmotic screws, which have five times greater tensile and rotational strength than suture fixation alone [117, 118].

Fibular shaft: High fibular fractures such as stage III or stage IV pronation-external rotation injuries according to the Lauge-Hansen classification system should be managed surgically (with plate fixation). Moreover, the underlying complex dislocation mechanism may also have caused other bony injuries (fractures of the medial malleolus) and/or ligamentous injuries (of the syndesmosis or the medial and/or lateral capsular ligaments) [119]. Distal fibula: Isolated fractures of the lateral malleolus are divided into stable and unstable fractures. Stable fractures occur at the level of the syndesmosis (Type B fractures according to the Weber classification) or stage II supination-external rotation fractures according to the Lauge-Hansen classification system [120–123]. A fracture of the lateral malleolus is considered stable if there is no fibular shortening, no fracture displacement > 2 mm, and no articular malalignment, and the posterior syndesmosis is intact [121, 122]. Stable lateral malleolar fractures can be treated nonoperatively, for example by immobilisation with a plaster splint or an orthosis. Other types of fractures must be managed surgically.

The type of fixation also depends on concomitant soft-tissue injuries (contusion, swelling, compartment syndrome) [124]. If possible and regardless of the severity of the other injuries, external fixation should initially be used for the management of higher-grade soft-tissue damage or complex types of fractures (e.g. fracture-dislocations) in order to prevent neurovascular damage [125]. In patients with lateral malleolar fractures that are stable or have been stabilised by fixation, follow-up treatment that includes early functional therapy and weight-bearing was associated with a significant improvement in the range of movement at the ankle and a shorter period of rehabilitation [126].

Isolated lower-extremity injuries should be initially managed with an external fixator in the damage control setting. External fixation should be liberally used as a damage control procedure in all “borderline” patients, especially in patients with concomitant traumatic brain injury or with a lack of improvement in haemodynamic and respiratory parameters in spite of extensive initial resuscitation room treatment and intensive care (based on the non-normalisation of lactate levels and non-improvement in ventilatory parameters) [127, 128].

### Multiple fractures of the lower extremity

The management strategy for **multiple fractures of the femoral shaft and the lower leg** in polytraumatised patients is a problem that has not yet been conclusively addressed. Although the reported incidence of 2–7% is indicative of the clinical significance of multiple fractures of the femoral shaft and the lower leg, there is a paucity of data in the literature. Most studies are based on retrospective clinical data (*n* = 42, 4–222 patients) and case reports (*n* = 29). These studies investigated mortality as their primary outcome as well as a wide variety of secondary outcomes such as complication rates, length of stay, and concomitant injuries. Whereas the vast majority of authors are of the opinion that patients benefit from an early stabilisation of these fractures, the technique and timing of fracture stabilisation remain controversial. The only prospective study that has so far been published revealed a high rate of pulmonary complications in patients who underwent multiple intramedullary nailing (62.5%) compared to patients with one intramedullary procedure (8.2%) [129]. As a consequence of these results, the author recommended a staged treatment strategy. By contrast, analyses of retrospective data did not detect an increased risk of pulmonary complications, e.g. pulmonary (fat) embolism, after multiple intramedullary nailing. Other authors reported a shorter period of recovery and lower complication rates in (paediatric) patients who underwent surgical stabilisation. They advocated primary definitive stabilisation. In conclusion, early definitive surgical stabilisation is increasingly favoured in the literature. The technique and timing of surgical stabilisation, however, remain a subject of controversy. For this reason, the best possible recommendation is to adopt a risk-adapted approach, similar to that used in the surgical management of isolated lower-extremity injuries (see above) [127, 128, 130]. The majority of severely injured patients who are haemodynamically stable within 24 h can then be safely managed with primary (multiple) intramedullary nailing. Haemodynamically unstable patients as well as patients with haemorrhagic shock or a combination of several severe injuries should be initially managed with an external fixator in the damage control setting. External fixation, as a damage control procedure, should be liberally used in all “borderline” patients, especially in patients with concomitant traumatic brain injury or with a lack of improvement in haemodynamic and respiratory parameters in spite of extensive initial resuscitation room treatment and intensive care (based, for example, on the non-normalisation of lactate levels and non-improvement in ventilatory parameters).

Since decisions on the management of both isolated and multiple lower-extremity long-bone fractures in polytraumatised patients are clinically important and must often be made in everyday clinical practice, further prospective studies with an appropriate design are urgently needed to identify the best treatment approaches.

### Definitive care for femoral shaft fractures in polytraumatised patients

Surgical stabilisation is considered the standard treatment for femoral shaft fractures (see recommendations 1. and2.). Indications for emergency surgery are open fractures, fractures with vascular and/or nerve injuries, and fractures with compartment syndrome. Early definitive fixation is the preferred treatment for haemodynamically stable patients. Most authors consider intramedullary nailing to be the gold standard [49–52]. Proponents of this technique argue that intramedullary nailing permits early weight-bearing.

Neudeck et al. [53], however, showed in a retrospective study on 255 polytraumatised patients with femoral fractures that only 29% of these patients were able to benefit from early weight-bearing after primary intramedullary nailing when the severity of injury, the extent of injury, and the clinical course were taken into consideration. The choice of the primary surgical technique (nailing versus plate fixation) in polytraumatised patients thus remains controversial among some authors [54–62]. Bone et al. [55] reported that the incidence of pulmonary complications solely depended on the patient’s chest injury and not on the technique of femoral fracture stabilisation (nail or plate). In a retrospective study on 217 patients who underwent intramedullary nailing with reaming for a femoral fracture and 206 patients who were managed with a plate, Bosse et al. [56] found no difference in the occurrence of adult respiratory distress syndrome (ARDS) between polytraumatised patients with a thoracic injury and those without a thoracic injury. In another retrospective study, Aufmkolk et al. [54] too reported that primary plate fixation of femoral fractures did not increase mortality and morbidity in patients with or without thoracic injuries (AIS thorax ≥ 3). Several animal models, including an animal study by Wozasek et al. [63], did not detect a significant difference in pulmonary and/or haemodynamic effects between intramedullary nailing and plate fixation. It is undisputed that fat embolism occurs as a result of an intramedullary pressure increase during nailing. Many clinical and animal studies demonstrated this effect, especially by echocardiography [64]. Whether or not this is clinically relevant remains unclear, as does the question of whether reamed or unreamed intramedullary nailing should be preferred. Several prospective randomised studies compared reamed and unreamed intramedullary nailing and found no differences in ARDS rates, pulmonary complications, and survival rates [65, 66].

Primary intramedullary nailing is considered contraindicated in haemodynamically stable patients with open grade III femoral fractures and vascular involvement [53, 67, 68]. In these cases, other procedures such as external fixation can be used to stabilise a fracture [131].

Femoral shaft fractures are associated with good callus formation and a low rate of complications [110]. Ligamentous injuries in the knee joint occur in 10–20% of femoral shaft fractures. Possible complications are bleeding, infection, wound healing problems, avascular femoral head necrosis, non-union, rotational deformity, limited range of motion, thrombosis, and embolism.

Patel et al. were able to show that definitive fixation should be performed as early as possible and that there was no difference in outcome between surgery during daytime working hours and surgery during nighttime hours [132].

### Treatment of lower-extremity dislocations in polytrauma patients

There are no controlled studies that address the management of knee dislocations specifically in multiply injured patients. The studies referred to below investigated patients with isolated knee dislocations as well as polytraumatised patients with knee dislocations. The highest priority must be given to possible vascular injuries (popliteal artery) that require treatment. Green and Allen [133] analysed 245 knee dislocations and found that vascular injuries were present in 32% of these patients. Of the patients who did not undergo vascular repair within eight hours from the time of injury, 86% had an amputation and two thirds of the remaining patients had ischaemic changes. Compartment release surgery is recommended for patients who are not treated within a period of six hours and for patients with an impending compartment syndrome.

The knee should be reduced as early as possible in haemodynamically stable or unstable patients with multiple injuries. If closed reduction is unsuccessful, the dislocated joint is reduced in an open procedure [134]. In patients who underwent planned conservative treatment and in patients who underwent planned early anterior cruciate ligament reconstruction, external fixation and transfixation with Steinmann pins or a brace or plaster cast may be used to ensure that the joint stays reduced. According to expert opinion, external fixation has advantages over other methods [135]. MRI-compatible implants should be used to allow imaging to be performed after reduction and temporary fixation. Ligamentous injuries after knee dislocations can be treated either surgically or nonoperatively. In a meta-analysis, Dedmond and Almekinders [136] compared the clinical outcomes from twelve retrospective and three prospective studies involving 132 surgically treated and 74 non-operatively treated knee dislocations. The surgical group had significantly better outcomes than the non-operative group in terms of range of motion (123 degrees versus 108 degrees), Lysholm scores (85.2 versus 66.5), and flexion contracture (0.5 degrees versus 3.5 degrees). The groups had not been randomised, and the indications for surgical or nonoperative treatment were not reported. Two other retrospective studies also demonstrated that surgical treatment was superior to nonoperative management [137, 138].

Injuries to the cruciate ligaments after knee dislocations can be managed surgically by direct suture repair or ligament replacement. Mariani et al. [139] conducted a retrospective study on a small number of patients with knee dislocations and reported that anterior and posterior cruciate ligament reconstruction using patellar tendon or semitendinosus tendon grafts was superior to direct suture repair in terms of stability and range of motion.

There are no controlled studies that address the management of hip dislocations specifically in multiply injured patients. A review found an incidence of less than 1% in polytraumatised patients [140]. Early diagnosis and reduction play an important role in outcome [141–143]. See recommendation 4.

### Antibiotic prophylaxis in the management of closed and open lower-extremity fractures

In patients with open fractures, bacterial contamination is present preoperatively in 48–60% of all wounds and in 100% of severe wounds [144].

Use of antibiotics in the management of closed fractures.

During the surgical treatment of closed fractures, the administration of prophylactic antibiotics (usually a single-dose long-acting first-generation cephalosporin) is generally recommended in procedures involving the implantation of foreign materials [145, 146]. The available evidence (level 1) on the management of femoral neck fractures demonstrates that perioperative prophylactic antibiotics significantly reduce postoperative wound infections [145, 147–149]. A Cochrane review that was conducted in 2003 and included data from 22 studies with 8307 patients showed that preoperative single-dose antibiotics significantly reduced postoperative wound infections as well as infections of the urogenital and respiratory tracts in patients who underwent surgery for long-bone fractures. Neither the Cochrane review by Gillespie et al. [150] nor a meta-analysis by Slobogean et al. [151] demonstrated advantages of multiple-dose over single-dose antibiotic regimens.

## Use of antibiotics in the management of open fractures

There is sufficient evidence to suggest that antimicrobial prophylaxis should be used in the management of open fractures. The Eastern Association for the Surgery of Trauma (EAST) guidelines recommend that antibiotic coverage directed at Gram-positive organisms be initiated as soon as possible in addition to thorough wound debridement [152, 153]. For Gustilo grade III fractures, additional coverage for Gram-negative organisms should be given. High-dose penicillin should be added in the presence of farm-related injuries in order to prevent potential clostridial contamination. Treatment should be continued until 24 h after primary soft-tissue coverage of the wound. For grade III fractures, antibiotics should be continued for 72 h after injury or not more than 24 h after soft-tissue coverage has been achieved [144]. In line with other studies [154], Dellinger et al. [155], who analysed data from 248 patients, found no significant difference in infection rates depending on the duration of preventive antibiotic administration (one day versus five days of therapy) [156, 157]. In the literature, the use of local antibiotic carriers, such as antibiotic-impregnated polymethyl methacrylate (PMMA) beads, in addition to systemic intravenous antibiotics is increasingly favoured for prophylactic purposes in the management of major open injuries with severe contamination [156, 158, 159]. The specific indications as well as the type and timing of local administration, however, remain controversial [154, 160–162]. See recommendation 5.

### Timing and treatment of vascular injuries of the lower extremities

There is a paucity of robust data on the incidence of arterial and venous injuries of the lower extremities in polytraumatised patients. The patient groups that were investigated in the international literature vary widely in terms of the severity, mechanism and location of their vascular (and other) injuries as well as in the quality of preoperative diagnostic evaluation and management [163–167]. Morphological changes to vessels depending on the type of injury and the role of these changes in treatment have been described in detail [168].

The treatment recommendations presented here are largely based on the knowledge and experience of experts who investigated specific patient groups and published their results and conclusions. Only one controlled randomised study is available [169]. The published recommendations come from a variety of subspecialties of trauma surgery and vascular surgery and allow only limited conclusions to be drawn on the management of severe lower-extremity injuries with vascular involvement in polytraumatised patients. For this reason, the approach to treatment must be individualised to the patient.

If possible, depending on overall injury severity, the surgical management of arterial and venous injuries of the lower extremities should be performed as soon as possible, i.e. immediately after the treatment of life-threatening injuries. This also applies to polytrauma patients. There is no consensus in the literature on whether fracture stabilisation should precede vascular reconstruction or vice versa. Temporary solutions are discussed as well (e.g. primary use of a shunt to maintain perfusion, fracture stabilisation, and definitive vascular reconstruction at a later stage, or damage control procedures until the restoration of physiological parameters) [170–177]. Primary vascular exploration and, if necessary, immediate vascular repair should be performed in patients with complex trauma that is associated with a high probability of vascular injury [169]. The resources, surgical principles and techniques that are available for this purpose are the same as those that are used in the management of non-traumatic arterial and venous conditions and are sometimes used in additional indications.

Injuries to the iliac and femoral arteries should be reconstructed and can usually be easily accessed. Isolated arterial injuries of the lower leg may be treated by ligation if the other major distal arteries are confirmed to be patent. Injuries to at least two vessels almost always lead to critically impaired perfusion that requires primary revascularisation. Combined arterial and venous injuries are associated with increased amputation rates. For this reason, venous reconstruction should be performed liberally in the management of combined injuries [164, 165, 178]. In descending order, the following options should be used in the management of arterial injuries to the lower extremities: direct suture repair, anastomosis to restore continuity, patch angioplasty (using autologous or synthetic material), or bypass grafting (using autologous, synthetic or composite grafts) [167, 179]. In descending order, the following options should be used in the management of venous injuries to the lower extremities: patch repair, autologous venous interposition grafts, polytetrafluoroethylene (PTFE) grafts, or primary ligation [180–186].

Fasciotomy should be performed early and, where necessary, should be carried out before vascular reconstruction [164, 187].

Endovascular procedures are a further option for managing arterial injuries of the lower extremities and can also be used in polytraumatised patients. Established techniques that are used at proximal extremity locations (coils, covered stents) can also be employed at peripheral sites in individual cases. Temporary revascularisation too can be performed prior to definitive surgical management [188–191]. See recommendation 6.

### Treatment of compartment syndrome

Compartment syndrome is not uncommon in association with lower-extremity long-bone fractures, especially with those involving the tibia. It can lead to disastrous consequences within a few hours and therefore requires early decompression (fasciotomy) during fracture stabilisation. Van den Brand et al. [192] even reported that prophylactic fasciotomy appeared to be superior to early therapeutic decompression. Early diagnosis of compartment syndrome is essential since irreversible damage to muscles and nerves can occur within eight hours or less [193]. Diagnosis is primarily based on clinical criteria [194]. Normal skin colour and temperature and the presence of distal pulses [193–196] do not exclude compartment syndrome. Pain, which is the cardinal symptom of compartment syndrome, as well as pain with muscle stretching and sensation tests cannot be used in polytraumatised patients who are usually unconscious or are receiving sedation and analgesia. For this reason, Rowland et al. [197] suggested that a pressure measuring device should be used for the definitive diagnosis in unconscious patients. An intracompartmental pressure of more than 30 mmHg in unconscious patients and a differential pressure (diastolic minus intracompartmental pressure) of more than 30 mmHg in patients with hypotension are considered the critical thresholds and indications for fasciotomy [193, 195, 196, 198, 199]. Once the diagnosis of compartment syndrome is made, immediate fasciotomy (emergency procedure) is indicated [193–196]. At the lower leg, all four muscle compartments should be opened. Prognosis depends on the overall injury pattern and is most favourable in patients with isolated compartment syndrome without a fracture. If a fracture is also present, stable fracture fixation should be performed in addition to fasciotomy. Intramedullary nailing [200, 201] is the preferred technique of fixation since, compared with other methods, it causes the least irritation of the soft tissues and does not require the placement of transfixation pins. In a meta-analysis, Bhandari et al. [202] assessed the relative risk of compartment syndrome in patients who were managed with either reamed or unreamed intramedullary nails. Although the difference was not significant (relative risk 0.45; 95% CI, 0.13 to 1.56), the authors concluded that reamed intramedullary nailing appeared to reduce the risk of compartment syndrome. The recommendation to act rapidly is based on experience rather than on specific studies on compartment syndrome in patients with multiple trauma. See recommendation 7.

### Amputation versus limb salvage in polytrauma patients

Severe injuries of the lower extremities can be a complex challenge in the management of patients with multiple injuries and may require critical decisions on amputation or limb salvage. The literature shows that a loss of neurologic function is correlated with delayed amputation and increased morbidity and mortality [203]. Early amputation should be considered if there is a loss of function and sensation in the foot and/or the lower extremity. Vice versa, limb salvage should be attempted if function and sensation in the foot and/or lower extremity are intact [203]. This means that amputation should be the preferred treatment for all patients with functional or neurologic deficits after a grade IIIc fracture and complete sciatic or tibial nerve disruption. There is no study indicating an advantage of limb salvage over amputation in patients with significant nerve disruption [204–206].

Vascular integrity increases the probability of limb salvage [207]. Perfusion should be restored as rapidly as possible. Ischaemic periods of more than six hours were reported to be correlated with irreversible nerve damage and loss of function [208, 209]. A necrotic extremity or part of an extremity should be amputated. A delay in amputation leads to a significant increase in the occurrence of sepsis, immobility, the number of required surgical procedures, mortality, and costs [204–206]. Objective criteria that can inform decisions on amputation or limb salvage have been published in a variety of studies [210–213]. No study, however, has so far defined an instrument that can give a guaranteed prediction on which to base the decision to amputate or attempt limb preservation. Scoring systems, e.g. the predictive salvage index, the Mangled Extremity Severity Score (MESS), the limb salvage score, or the Nerve Injury, Ischaemia, Soft-tissue Injury, Skeletal Injury, Shock and Age of Patient (NISSSA) score, can be used as adjuncts to clinical assessment. For this reason, it is absolutely necessary that treatment decisions be individualised to the patient and tailored to the injury. The decision to amputate or attempt limb salvage should never be based solely on a protocol or algorithm [214, 215]. In conclusion, primary and secondary amputation rates in patients with lower-extremity injuries (which cannot be predicted, for example, on the basis of scoring systems) thus depend on the number and location of concomitant arterial and venous injuries, nerve injuries, overall injury severity, and the extent of associated soft-tissue damage [164, 165, 167, 174, 178, 187, 216–224]. See recommendation 8.

### Relation to other studies

Compared with the previous version of the S3 Polytrauma Guideline, topics were restructured. Headings and subjects were consolidated to provide a clearer structure of the current guideline. Our recommendations are mainly applicable to Western European and similar trauma systems. Guidelines from other international societies which focus on non-orthopaedic trauma, including the EAST [59–61] and the World Society of Emergency Surgery (WJES) [62] guidelines, are based on a similar work-up. The validity of their findings and recommendations, however, is unclear for the situation in Germany (and similar countries). Furthermore, most of the previously mentioned guidelines are more than five years old and may require revisions. The recommendations from both this guideline and other guidelines should act as adjuncts to each other and may be used to optimise local protocols. Other parts of the updated S3 Polytrauma Guideline have been published as well.

## Limitations

Experimental and preclinical studies have been excluded from this review. Data pooling was not performed. For this reason, no pooled analysis addressing specific research questions was undertaken. Following the systematic literature search, a consensus meeting was performed during which proposed recommendations from the working group were discussed. A non-weighted voting procedure rather than a structured procedure, e.g. according to the Delphi approach, was performed.

## Implications for practice

The results of the literature review and the consensus recommendations are described in Table 5. An extrapolation of these findings and the integration of recommendations into local guidelines may be considered.

### Unanswered questions and future research

There is a clear lack of high-quality evidence since prospective and randomised studies are rare. For this reason, more prospective multi-centre studies are warranted. Since the level of evidence of all studies is reported as well, topics of interest can easily be extracted from this manuscript.

## Electronic supplementary material

Below is the link to the electronic supplementary material.

Supplementary Material 1

## Data Availability

No datasets were generated or analysed during the current study.

## Abbreviations

AHRQ: Agency for Healthcare Research and Quality
AIS: Abbreviated Injury Scale
ARDS: Adult respiratory distress syndrome
AWMF: Association of the Scientific Medical Societies in Germany
DCO: Damage control orthopaedics
EAST: Eastern Association for the Surgery of Trauma
GCS: Glasgow Coma Scale
GPP: Good (clinical) practice point
ISS: Injury Severity Score
LoE: level of evidence
MESS: Mangled Extremity Severity Score
NISSA: Nerve Injury, Ischaemia, Soft-tissue Injury, Skeletal Injury, Shock and Age of Patient
PICO: Population, Intervention, Comparison, Outcome
PMMA: Polymethyl methacrylate
PRISMA: Preferred Reporting Items for Systematic Reviews and Meta-Analyses
PTFE: Polytetrafluoroethylene
RCT: Randomised controlled trial
RoB: Risk of bias
WJES: World Society of Emergency Surgery
