# Supplementary material for: Initial surgical management of injuries to the lower extremities in patients with multiple and/or severe injuries – A systematic review and clinical practice guideline update
Source: Eur J Trauma Emerg Surg. 2024 Nov 5;50(6):3329–50. doi: 10.1007/s00068-024-02662-0 (PMC11666759; doi:10.1007/s00068-024-02662-0)
Supplement: Supplementary file 1 — Supplementary Material 1 [file 68_2024_2662_MOESM1_ESM.docx]

3.10 Lower extremities

# S1 PICO Questions

|  | Population | Intervention(s) | Control(s) | Outcome(s) |
| --- | --- | --- | --- | --- |
| 1 | Target population, with isolated and multiple shaft fractures of long bones of the lower extremities. | primary-definitive, primary-temporary or secondary-definitive osteosynthetic treatment | Primary-definitive, primary-temporal or secondary-definitive osteosynthetic treatment or no osteosynthesis | Patient-relevant outcomes |
| 2 | Target population, with isolated closed shaft fractures of the tibia | Primary-temporary stabilisation with plaster cast | No primary temporal stabilisation by plaster (osteosynthetic treatment) | Patient-relevant outcomes |
| 3 | Target population, with proximal femur fracture | Primary osteosynthetic stabilisation | No primary osteosynthetic treatment (cross-joint fixator ex.) | Patient-relevant outcomes |
| 4 | Target population, with proximal femur fracture | Interlocking intramedullary nailing | No locking intramedullary nailing (plate osteosynthesis) | Patient-relevant outcomes |
| 5 | Target population, with unstable distal femur fractures | Primary surgical stabilisation (non-definitive) | No primary surgical stabilisation (definitive osteosynthetic treatment) | Patient-relevant outcomes. |
| 6 | Target population, with knee dislocation | Early closed reduction, if closed reduction unsuccessful open reduction. Retention by external fixator or similar possible | Late reduction | Patient-relevant outcomes |
| 7 | Target population, with proximal tibia and tibial plateau fractures | Primary stabilisation by splinting | No primary stabilisation by splinting (operative fixation with osteosynthesis) | Patient-relevant outcomes |
| 8 | Target population, with proximal tibial plateau fractures | Surgical stabilisation (usually by intramedullary nailing) | Stabilisation by plaster | Patient-relevant outcomes |
| 9 | Target population, with distal tibial fractures including articular distal tibial fractures | Definitive osteosynthetic treatment | No definitive osteosynthetic treatment (primary treatment with external fixator or, if necessary, K-wires depending on localisation) | Patient-relevant outcomes |
| 10 | Target population, with ankle fractures | Primary stabilisation (surgical/non-surgical) | No primary stabilisation (operative/non-operative) | Patient-relevant outcomes |
| 11 | Target population, with open and closed lower limb fractures | Perioperative antibiotic prophylaxis | No perioperative antibiotic prophylaxis | Postoperative wound infections, patient-relevant outcomes |
| 12 | Target population, with lower limb vascular injuries | Early surgical treatment of vascular injuries as soon as vital signs permit (even before reduction of fractures) | Late treatment of vascular injuries | Amputation rate, patient-relevant outcomes |
| 13 | Target population, with lower limb compartment syndrome (and associated fracture) | Early (possibly even prophylactic) fasciotomy for compartmental decompression. If necessary, followed by osteosynthesis by intramedullary nailing. | Late fasciotomy | Irreversible Schäden, Patientenrelevante Outcomes |
| 14 | Target population, with severe lower limb injuries | Amputation | Limb preservation | Patientenrelevante Outcomes |

^§^ new question

# S2 Literature Search

| Suchstrategie 2021, MEDLINE (via Ovid) Datum: 06.05.2021 1.717 Treffer |
| --- |
| 1. exp Lower Extremity/in  2. ((lower extremit* or lower limb* or leg* or thigh or femur or femoral or tibia* or fibula* or knee* or ankle or malleolus or calcaneus or talus) adj2 (trauma* or injur* or fracture* or luxat*)).ti,ab,kf.  3. 1 or 2  4. exp Multiple Trauma/  5. (polytrauma* or trauma patient?).ti,ab,kf. or (severe adj2 shock).ti,ab,kf.  6. ((multiple or major or severe* or serious*) adj3 (trauma* or injur*)).ti,ab,kf.  7. ((blunt or penetrating) adj5 (trauma* or injur*)).ti,ab,kf.  8. (*Critical Care/ or *Emergencies/ or (life threatening or critical care or emergen*).ti,ab,kf.) and (trauma* or injur*).ti,ab,kf.  9. 4 or 5 or 6 or 7 or 8  10. 3 and 9  11. exp animals/ not humans.sh.  12. 10 not 11  13. (comment or editorial or letter).pt. or case report*.mp.  14. 12 not 13  15. limit 14 to dt=20090101-20210315  16. exp Fracture Fixation/ or exp Fracture Fixation, Internal/ or exp fracture fixation, intramedullary/ or *surgical fixation devices/ or *orthopedic fixation devices/ or exp external fixators/ or exp internal fixators/ or exp Compartment Syndromes/ or (surger* or surgical or operation* or operative or fixation or fixator? or stabili* or nail? or plate? or screw? or wire? or pin? or osteosynthe* or compartment or antibio* prophyla* or reduction or retain or cast or amputation).ti,ab,kf.  17. 15 and 16 |
| Suchstrategie 2021, Embase (via Elsevier) Datum: 06.05.2021 730 Treffer |
| #1 'lower limb'/exp OR 'leg injury'/exp  #2 (("lower extremit*" OR "lower limb*" OR leg* OR thigh OR femur OR femoral OR tibia* OR fibula* OR knee* OR ankle or malleolus or calcaneus or talus) NEXT/2 (trauma* OR injur* OR fracture* OR luxat*)):ti,ab,kw  #3 #1 or #2  #4 'multiple trauma'/exp  #5 (polytrauma* OR "trauma patient?"):ti,ab,kw OR (severe NEXT/2 shock):ti,ab,kw  #6 ((multiple OR major OR severe* OR serious*) NEXT/3 (trauma* OR injur*)):ti,ab,kw  #7 ((blunt OR penetrating) NEXT/5 (trauma* OR injur*)):ti,ab,kw  #8 ('intensive care'/mj OR 'emergency'/mj OR ("life threatening" OR "critical care" OR emergen*):ti,ab,kw) AND (trauma* OR injur*):ti,ab,kw  #9 #4 OR #5 OR #6 or #7 OR #8  #10 #3 AND #9  #11 'animals'/exp NOT 'humans'/de  #12 #10 NOT #11  #13 (comment OR editorial OR letter):it OR "case report*":ti,ab,kw  #14 #12 NOT #13  #15 [1-1-2009]/sd NOT [16-3-2021]/sd  #16 #14 AND #15  #17 'fracture fixation'/exp OR 'osteosynthesis'/exp OR 'intramedullary nailing'/exp OR 'orthopedic fixation device'/exp OR 'external fixator'/exp OR 'internal fixator'/exp OR 'compartment syndrome'/exp OR (surger* OR surgical OR operation* OR operative OR fixation OR fixator? OR stabili* OR nail? OR plate? OR screw? OR wire? OR pin? OR osteosynthe* OR compartment OR “antibio* prophyla*” or reduction or retain or cast or amputation):ti,ab,kw  #18 #16 AND #17  #19 [embase]/lim  #20 #18 AND #19  #21 embase NOT (embase AND medline)  #22 #20 AND #21  #23 #22 AND ('article'/it OR 'article in press'/it OR 'erratum'/it OR 'review'/it) |

# S3 Excluded Studies

| **Reference** | **Reason for exclusion** |
| --- | --- |
| *Erratum: Timing of definitive treatment of femoral shaft fractures in patients with multiple injuries: A systematic review of randomized and nonrandomized trials (Journal of Trauma and Acute Care Surgery (2012) 73 (1046-1063)).* Journal of Trauma and Acute Care Surgery, 2012. **73**(6): p. 1631. | STUDY TYPE |
| *Orthopedic surgery and trauma.* International Angiology, 2013. **32**(2): p. 140-163. | POPULATION |
| Achten, J., et al., *UK Fixation of Distal Tibia Fractures (UK FixDT): protocol for a randomised controlled trial of 'locking' plate fixation versus intramedullary nail fixation in the treatment of adult patients with a displaced fracture of the distal tibia.* BMJ Open, 2015. **5**(9): p. e009162. | POPULATION |
| Alarhayem, A.Q., et al., *Impact of time to repair on outcomes in patients with lower extremity arterial injuries.* Journal of Vascular Surgery, 2019. **69**(5): p. 1519-1523. | POPULATION |
| Andruszkow, H., et al., *Surgical strategies in polytraumatized patients with femoral shaft fractures - comparing a German and an Australian level I trauma centre.* Injury, 2013. **44**(8): p. 1068-72. | STUDY TYPE |
| Bedes, L., et al., *External fixation of distal femoral fractures in adults' multicentre retrospective study of 43 patients.* Orthopaedics & traumatology, surgery & research, 2014. **100**(8): p. 867-72. | POPULATION |
| Burkhardt, G.E., et al., *Outcomes of selective tibial artery repair following combat-related extremity injury.* Journal of Vascular Surgery, 2010. **52**(1): p. 91-6. | POPULATION |
| Byrne, J.P., et al., *Timing of femoral shaft fracture fixation following major trauma: A retrospective cohort study of United States trauma centers.* PLoS Medicine / Public Library of Science, 2017. **14**(7): p. e1002336. | POPULATION |
| Cardozo, R.T., et al., *Treatment of Fractures of the Tibial Diaphysis Using External Fixator Compared with Locked Intramedullary Nails.* Revista Brasileira de Ortopedia, 2013. **48**(2): p. 137-144. | POPULATION |
| Cimbanassi, S., et al., *Orthopedic injuries in patients with multiple injuries: Results of the 11th trauma update international consensus conference Milan, December 11, 2017.* The Journal of Trauma and Acute Care Surgery, 2020. **88**(2): p. e53-e76. | STUDY TYPE |
| Dousa, P., et al., *[Ipsilateral fractures of the proximal femur and the femoral shaft].* Acta Chirurgiae Orthopaedicae et Traumatologiae Cechoslovaca, 2010. **77**(5): p. 378-88. | STUDY TYPE |
| Duyos, O.A., et al., *Management of Open Tibial Shaft Fractures: Does the Timing of Surgery Affect Outcomes?* Journal of the American Academy of Orthopaedic Surgeons, 2017. **25**(3): p. 230-238. | STUDY TYPE |
| El-Menyar, A., et al., *Early versus late intramedullary nailing for traumatic femur fracture management: meta-analysis.* Journal of Orthopaedic Surgery, 2018. **13**(1): p. 160. | STUDY TYPE |
| Fitschen-Oestern, S., et al., *Missed foot fractures in multiple trauma patients.* BMC Musculoskeletal Disorders, 2019. **20**(1): p. 121. | ACCORDING TO NO RECOMMENDATION |
| Futchko, J., et al., *A propensity-matched analysis of contemporary outcomes of blunt popliteal artery injury.* Journal of Vascular Surgery, 2020. **72**(1): p. 189-197. | POPULATION |
| Garcia Renedo, R.J., et al., *[Benefit of the stabilization of long-bone fractures in polytraumatized patients].* Acta Ortopedica Mexicana, 2010. **24**(1): p. 3-7. | LANGUAGE |
| Gasser, B., et al., *Damage control surgery - experiences from a level I trauma center.* BMC Musculoskeletal Disorders, 2017. **18**(1): p. 391. | STUDY TYPE |
| Giannoudis, P.V., et al., *Management, complications and clinical results of femoral head fractures.* Injury, 2009. **40**(12): p. 1245-51. | POPULATION |
| Grigorian, A., et al., *Decreased National Rate of below the Knee Amputation in Patients with Popliteal Artery Injury.* Annals of Vascular Surgery, 2019. **57**: p. 1-9. | POPULATION |
| Hartsock, L.A., et al., *Randomized prospective clinical trial comparing reamer irrigator aspirator (RIA) to standard reaming (SR) in both minimally injured and multiply injured patients with closed femoral shaft fractures treated with reamed intramedullary nailing (IMN).* Injury, 2010. **41**: p. S94-8. | ACCORDING TO NO RECOMMENDATION |
| hi, R.R., et al., *Optimal timing of femur fracture stabilization in polytrauma patients: A practice management guideline from the Eastern Association for the Surgery of Trauma.* The Journal of Trauma and Acute Care Surgery, 2014. **77**(5): p. 787-795. | STUDY TYPE |
| Hierholzer, C., A. Woltmann, and V. Buhren, *[Therapy of femur shaft fractures].* Zeitschrift fur Orthopadie & Unfallchirurgie, 2009. **147**(1): p. 91-115, quiz 116-7. | STUDY TYPE |
| Husebye, E.E., et al., *Intramedullary nailing of femoral shaft fractures in polytraumatized patients. a longitudinal, prospective and observational study of the procedure-related impact on cardiopulmonary- and inflammatory responses.* Scandinavian Journal of Trauma, Resuscitation & Emergency Medicine, 2012. **20**: p. 2. | STUDY TYPE |
| Jafree, S.B.H., et al., *Comparison of two techniques of interlocking intramedullary nailing in fractures of Tibia.* Pakistan Journal of Medical and Health Sciences, 2013. **7**(1): p. 231-235. | POPULATION |
| Jupiter, D.C., et al., *Incidence and risk factors for amputation in foot and ankle trauma.* Journal of Foot & Ankle Surgery, 2012. **51**(3): p. 317-22. | STUDY TYPE |
| Karapinar, L., et al., *Leg length discrepancies in adult femoral shaft fractures treated with intramedullary nailing.* Ulusal Travma ve Acil Cerrahi Dergisi = Turkish Journal of Trauma & Emergency Surgery: TJTES, 2009. **15**(3): p. 256-61. | POPULATION |
| Kauvar, D.S., M.R. Sarfati, and L.W. Kraiss, *National trauma databank analysis of mortality and limb loss in isolated lower extremity vascular trauma.* Journal of Vascular Surgery, 2011. **53**(6): p. 1598-603. | POPULATION |
| Kauvar, D.S., et al., *Early Fasciotomy and Limb Salvage and Complications in Military Lower Extremity Vascular Injury.* Journal of Surgical Research, 2021. **260**: p. 409-418. | POPULATION |
| Kugelman, D.N., et al., *Knee Stiffness After Tibial Plateau Fractures: Predictors and Outcomes (OTA-41).* Journal of Orthopaedic Trauma, 2018. **32**(11): p. e421-e427. | POPULATION |
| Kumaravel, S., et al., *Efficiency of fibular plating in distal third leg fractures in correcting valgus of the distal tibial fragment treated with interlocking nailing.* Research Journal of Pharmaceutical, Biological and Chemical Sciences, 2019. **10**(5): p. 1-5. | POPULATION |
| Langfitt, M.K., et al., *Distal locking using an electromagnetic field-guided computer-based real-time system for orthopaedic trauma patients.* Journal of Orthopaedic Trauma, 2013. **27**(7): p. 367-72. | POPULATION |
| Large, T.M., et al., *Does perioperative systemic infection or fever increase surgical infection risks after internal fixation of femur and tibia fractures in an intensive care polytrauma unit?* The Journal of Trauma and Acute Care Surgery, 2013. **75**(4): p. 664-8. | STUDY TYPE |
| Lichte, P., et al., *Are bilateral tibial shaft fractures associated with an increased risk for adverse outcome?* Injury, 2014. **45**(12): p. 1985-9. | STUDY TYPE |
| Lin, D., et al., *Emergent surgical reduction and fixation for Pipkin type I femoral fractures.* Orthopedics, 2013. **36**(6): p. 778-82. | POPULATION |
| Lodde, M.F., et al., *Union rates and functional outcome of double plating of the femur: systematic review of the literature.* Archives of Orthopaedic & Trauma Surgery, 2021. **23**: p. 23. | STUDY TYPE |
| Mao, Y., et al., *Association of surgical timing and operative outcomes in multiple ligaments knee injuries: A meta-analysis.* Chinese Journal of Evidence-Based Medicine, 2019. **19**(4): p. 418-423. | LANGUAGE |
| Matsumoto, S., et al., *Outcomes Comparison Between Ligation and Repair after Major Lower Extremity Venous Injury.* Annals of Vascular Surgery, 2019. **54**: p. 152-160. | ACCORDING TO NO RECOMMENDATION |
| Mioc, M.L., et al., *Extra-articular distal tibia fractures-controversies regarding treatment options. A single-centre prospective comparative study.* International Orthopaedics, 2018. **42**(4): p. 915-919. | POPULATION |
| Nahm, N.J. and H.A. Vallier, *Timing of definitive treatment of femoral shaft fractures in patients with multiple injuries: a systematic review of randomized and nonrandomized trials.* The Journal of Trauma and Acute Care Surgery, 2012. **73**(5): p. 1046-63. | ACCORDING TO NO RECOMMENDATION |
| Nicholas, B., et al., *Borderline femur fracture patients: early total care or damage control orthopaedics?* ANZ Journal of Surgery, 2011. **81**(3): p. 148-53. | STUDY TYPE |
| O'Toole, R.V., et al., *Local Antibiotic Therapy to Reduce Infection After Operative Treatment of Fractures at High Risk of Infection: A Multicenter, Randomized, Controlled Trial (VANCO Study).* Journal of Orthopaedic Trauma, 2017. **31**: p. S18-S24. | STUDY TYPE |
| Odl, R.M. , and A.H. Schmidt, *Compartment syndrome ultrafiltration catheters: report of a clinical pilot study of a novel method for managing patients at risk of compartment syndrome.* Journal of Orthopaedic Trauma, 2011. **25**(6): p. 358-65. | ACCORDING TO NO RECOMMENDATION |
| Oh, C.W., et al., *Minimally invasive plate osteosynthesis of subtrochanteric femur fractures with a locking plate: a prospective series of 20 fractures.* Archives of Orthopaedic & Trauma Surgery, 2009. **129**(12): p. 1659-65. | STUDY TYPE |
| Olson, J.J., et al., *The Judicious Use of Early Fixation of Closed, Complete Articular Pilon Fractures is Not Associated with Increased Risk of Deep Infection or Wound Complications.* Journal of Orthopaedic Trauma, 2020. **30**: p. 30. | POPULATION |
| Ondari, J.N., et al., *Unblinded randomized control trial on prophylactic antibiotic use in gustilo II open tibia fractures at Kenyatta National Hospital, Kenya.* Injury, 2016. **47**(10): p. 2288-2293. | POPULATION |
| Perkins, Z.B., et al., *Meta-analysis of prognostic factors for amputation following surgical repair of lower extremity vascular trauma.* British Journal of Surgery, 2015. **102**(5): p. 436-50. | STUDY TYPE |
| Polzer, H., et al., *Diagnosis and treatment of acute ankle injuries: development of an evidence-based algorithm.* Orthopedic Reviews, 2012. **4**(1): p. e5. | STUDY TYPE |
| Potter, H.A., et al., *Endovascular versus open repair of isolated superficial femoral and popliteal artery injuries.* Journal of Vascular Surgery, 2021. **5**: p. 05. | FULL TEXT NOT OBTAINABLE |
| Powell, S., *A comparison of two interventions in the treatment of severe ankle sprains and lateral malleolar avulsion fractures.* Emergency Nurse, 2019. **27**(5): p. 23-30. | POPULATION |
| Reavley, P., et al., *Randomised trial of the fascia iliaca block versus the '3-in-1' block for femoral neck fractures in the emergency department.* Emergency Medicine Journal, 2015. **32**(9): p. 685-9. | POPULATION |
| Renken, F., et al., *Early functional results after hemiarthroplasty for femoral neck fracture: a randomized comparison between a minimal invasive and a conventional approach.* BMC Musculoskeletal Disorders, 2012. **13**: p. 141. | POPULATION |
| Rixen, D., et al., *Protocol for a randomized controlled trial on risk adapted damage control orthopedic surgery of femur shaft fractures in multiple trauma patients.* Trials [Electronic Resource], 2009. **10**: p. 72. | STUDY TYPE |
| Rodriguez-Merchan, E.C., L. Moraleda, and P. Gomez-Cardero, *Injuries associated with femoral shaft fractures with special emphasis on occult injuries.* Archives of Bone & Joint Surgery, 2013. **1**(2): p. 59-63. | STUDY TYPE |
| Rollo, G., et al., *Surgical treatment of multifragmentary segmental femur shaft fractures with ORIF and bone graft versus MIPO: a prospective control-group study.* Medicinski Glasnik Ljekarske Komore Zenickodobojskog Kantona, 2020. **17**(2): p. 498-508. | ACCORDING TO NO RECOMMENDATION |
| Sahota, O., et al., *Femoral nerve block Intervention in Neck of Femur fracture (FINOF): study protocol for a randomized controlled trial.* Trials [Electronic Resource], 2014. **15**: p. 189. | STUDY TYPE |
| Sangkomkamhang, T., et al., *Incidence and risk factors for complications after definitive skeletal fixation of lower extremity in multiple injury patients: a retrospective chart review.* F1000Research, 2018. **7**: p. 612. | STUDY TYPE |
| Southeast Fracture, C., *LCP Versus LISS in the Treatment of Open and Closed Distal Femur Fractures: Does it Make a Difference?* Journal of Orthopaedic Trauma, 2016. **30**(6): p. e212-6. | ACCORDING TO NO RECOMMENDATION |
| Spering, C., et al., *It is time for a change in the management of elderly severely injured patients! An analysis of 126,015 patients from the TraumaRegister DGU<sup> R</sup>.* European Journal of Trauma & Emergency Surgery, 2020. **46**(3): p. 487-497. | STUDY TYPE |
| Streubel, P.N., P. Desai, and M. Suk, *Comparison of RIA and conventional reamed nailing for treatment of femur shaft fractures.* Injury, 2010. **41**: p. S51-6. | STUDY TYPE |
| Tahir, M.M., et al., *A Multicentre Randomized Controlled Trial Comparing Plating with Intramedullary Nailing for Extra-articular Distal Tibial Fractures.* Injury, 2021. **52**(1): p. 19-25. | POPULATION |
| Testa, G., et al., *Definitive Treatment of Femoral Shaft Fractures: Comparison between Anterograde Intramedullary Nailing and Monoaxial External Fixation.* Journal of Clinical Medicine, 2019. **8**(8): p. 28. | STUDY TYPE |
| Vestergaard, V., et al., *20-year trends of distal femoral, patellar, and proximal tibial fractures: a Danish nationwide cohort study of 60,823 patients.* Acta Orthopaedica, 2020. **91**(1): p. 109-114. | STUDY TYPE |
| Vicenti, G., et al., *Major concern in the multiligament-injured knee treatment: A systematic review.* Injury, 2019. **50**: p. S89-S94. | POPULATION |
| Willett, K., et al., *Close Contact Casting vs Surgery for Initial Treatment of Unstable Ankle Fractures in Older Adults: A Randomized Clinical Trial.* JAMA, 2016. **316**(14): p. 1455-1463. | POPULATION |
| Won, Y., et al., *Improved functional outcome after early reduction in Bosworth fracture-dislocation.* Journal of Foot & Ankle Surgery, 2019. **25**(6): p. 798-803. | POPULATION |
| Yao, C., et al., *Early results of reverse less invasive stabilization system plating in treating elderly intertrochanteric fractures: a prospective study compared to proximal femoral nail.* Chinese Medical Journal, 2011. **124**(14): p. 2150-7. | POPULATION |
| Yazdanpanah, P. and H. Mohammadi, *Short-term complications of hip bipolar hemiarthroplasty with anterior approach in patients with femoral neck fracture admitted to the emergency department of yasuj shahid beheshti hospital in 2016-2018.* Revista Latinoamericana de Hipertension, 2020. **14**(2): p. 150-153. | ACCORDING TO NO RECOMMENDATION |
| Zura, R.D., et al., *Timing of definitive fixation of severe tibial plateau fractures with compartment syndrome does not have an effect on the rate of infection.* Journal of Trauma-Injury Infection & Critical Care, 2010. **69**(6): p. 1523-6. | POPULATION |
| Akula, M., et al., *A meta-analysis of amputation versus limb salvage in mangled lower limb injuries--the patient perspective.* Injury, 2011. **42**(11): p. 1194-7. | STUDY TYPE |
| Bhatnagar, V., et al., *Retrospective study of cardiovascular disease risk factors among a cohort of combat veterans with lower limb amputation.* Vascular Health & Risk Management, 2019. **15**: p. 409-418. | POPULATION |
| Bosse, M.J., et al., *Assessment of Severe Extremity Wound Bioburden at the Time of Definitive Wound Closure or Coverage: Correlation With Subsequent Postclosure Deep Wound Infection (Bioburden Study).* Journal of Orthopaedic Trauma, 2017. **31**: p. S3-S9. | ACCORDING TO NO RECOMMENDATION |
| Doshi, P., et al., *Incidence of infection following internal fixation of open and closed tibia fractures in India (INFINITI): a multi-centre observational cohort study.* BMC Musculoskeletal Disorders, 2017. **18**(1): p. 156. | POPULATION |
| Dubrov, V.E., et al., *[Peculiarities of surgical treatment of wounded with combined thermomechanical injuries of the lower extremities under conditions of counter-terrorist operation].* Voenno-Meditsinskii Zhurnal, 2015. **336**(11): p. 27-37. | LANGUAGE |
| Ebrahimi, A., et al., *Early Reconstructions of Complex Lower Extremity Battlefield Soft Tissue Wounds.* World Journal of Plastic Surgery, 2017. **6**(3): p. 332-342. | STUDY TYPE |
| Eskridge, S.L., et al., *Association of Specific Lower Extremity Injuries With Delayed Amputation.* Military Medicine, 2019. **184**(5): p. e323-e329. | ACCORDING TO NO RECOMMENDATION |
| Jiang, M., et al., *Early intramedullary nailing of femoral shaft fracture on outcomes in patients with severe chest injury: A meta-analysis.* Scientific Reports, 2016. **6**: p. 30566. | STUDY TYPE |
| Joveniaux, P., et al., *Distal tibia fractures: management and complications of 101 cases.* International Orthopaedics, 2010. **34**(4): p. 583-8. | POPULATION |
| Lantry, J.M., V. Perumal, and C.S. Roberts, *Can patterns of segmental injuries of the foot and ankle predict amputation and disability?* Journal of Surgical Orthopaedic Advances, 2009. **18**(3): p. 134-8. | FULL TEXT NOT OBTAINABLE |
| Li, B., Y. Yang, and L.S. Jiang, *Plate fixation versus intramedullary nailing for displaced extra-articular distal tibia fractures: a system review.* European journal of orthopaedic surgery & traumatologie, 2015. **25**(1): p. 53-63. | STUDY TYPE |
| Liu, X., et al., *[Safety evaluation of secondary conversion from external fixation to internal fixation for open tibia fractures].* Chung-Kuo Hsiu Fu Chung Chien Wai Ko Tsa Chih/Chinese Journal of Reparative & Reconstructive Surgery, 2017. **31**(6): p. 665-669. | LANGUAGE |
| Liu, X.Y., et al., *Early intramedullary nailing for femoral fractures in patients with severe thoracic trauma: A systemic review and meta-analysis.* Chinese Journal of Traumatology, 2016. **19**(3): p. 160-3. | STUDY TYPE |
| Melcer, T., et al., *A comparison of health outcomes for combat amputee and limb salvage patients injured in Iraq and Afghanistan wars.* The Journal of Trauma and Acute Care Surgery, 2013. **75**(2): p. S247-54. | ACCORDING TO NO RECOMMENDATION |
| Perkins, Z.B., et al., *Meta-analysis of prognostic factors for amputation following surgical repair of lower extremity vascular trauma.* British Journal of Surgery, 2015. **102**(5): p. 436-450. | STUDY TYPE |
| Shashank Jain, S.N.S.K.S.G.A.M., *Clinical Outcome of Supracondylar Femoral Fractures Managed Locking Plate Osteosynthesis in a Rural Hospital.* Indian Journal of Forensic Medicine &amp; Toxicology, 2020. **14**(4): p. 6450-6456. | POPULATION |
| Singleton, J.A., et al., *Case suitability for definitive through knee amputation following lower extremity blast trauma: analysis of 146 combat casualties, 2008-2010.* Journal of the Royal Army Medical Corps, 2014. **160**(2): p. 187-90. | STUDY TYPE |
| Tekin, L., et al., *Comparison of quality of life and functionality in patients with traumatic unilateral below knee amputation and salvage surgery.* Prosthetics & Orthotics International, 2009. **33**(1): p. 17-24. | STUDY TYPE |

# S4 Evidence Table

##### In this chapter, the LoE was not downgraded.

##### Recommendation 3.67 & 3.69

| Study: Reference, aim, design, setting | Participants: selection criteria, characteristics | N Participants; Intervention (IG) vs. Control group (CG) | Main outcomes | Assessment: LoE, risk of bias; Conclusions |
| --- | --- | --- | --- | --- |
| Blair 2019  “Early Stabilization of Femur Fractures in the Setting of Polytrauma is Associated With Decreased Risk of Pulmonary Complications and Mortality” *J Surg Orthop Adv. Summer* 2019;28(2):137-143.  Study design  Comparative registry study  Aim of the study  The purpose of the study is to identify ideal timing of definitive fixation of femoral shaft fractures in the setting of polytrauma with regard to decreased risk of major systemic complications, mortality and lengths of hospital stay by using a large nationalized hospital database.  Setting  USA, 2009-2012 | Inclusion criteria   - Age 18-65 years - ICD-9 821.01 and 821.11 (closed and open femoral shaft fracture) - ISS >15 - Underwent femoral external or internal fixation - Time to surgery available   Characteristics  Age [y], mean ± SD  IG1: 32.3 ± 30.5  IG2: 33.9 ± 30.2, p=0.1208 IG3: 35.4 ± 52.2, p=0.1385 CG: 35.1 ± 30.2, p=0.0002  ISS, mean ± SD  IG1: 24.3 ± 23.9)  IG2: 26.2 ± 26.9, p=0.0048 IG3: 30.7 ± 62.9, p=0.0163 CG: 30.4 ± 49.3, p<0.0001  GCS, mean ± SD  IG1: 13.3 ± 17.2 IG2: 12.3 ± 15.6, p=0.0052) IG3: 11.9 ± 12.9, p=0.0091) CG: 11.1 ± 22.0, p<0.0001  Pulmonary contusion (ICD-9 861-20/21), (%)  IG1: 6.9  IG2: 2.2, p=0.0070 IG3: 1, p=0.0001) CG: 1, p<0.0001 | Participants  N=10,072 patients  Study groups  IG1: definitive fixation <24 hours (N=6,569)  IG2: definitive fixation 24-48 hours (N=1,327)  IG3: definitive fixation 48-72 hours (N=631)  CG: definitive fixation >72 hours (N=1545) | Univariate analysis time to surgery with reference category <24h  Hospital LOS, regression coefficient (SD)  0.07 (0.01), p<0.0001  ICU LOS, regression coefficient (SD)  0.04 (0.01), p<0.0001  Ventilator time, regression coefficient  (SD) 0.03 (0.01), p<0.0001  ARDS, OR (95% CI)  1.005 (1.003-1.007), p<0.0001  Major systemic complication, OR (95% CI)  1.005 (1.003-1.006), p<0.0001*  Mortality (in hospital), OR (95% CI)  1.001 (1.000-1.002), p=0.028  Multivariate analysis time to surgery 24-48h with reference category <24h  Hospital LOS, regression coefficient (SD)  2.6 (0.87), p=0.0037  ICU LOS, regression coefficient (SD)  NR, NS  Ventilator time, regression coefficient (SD)  2.35 (1.13), p=0.0422  ARDS, OR (95% CI)  1.50 (1.01-2.23), p=0.0431  Major systemic complication, OR (95% CI)  NR, NS*  Mortality (in hospital), OR (95% CI)  NR, NS  Multivariate analysis time to surgery 49-72h with reference category <24h  Hospital LOS, regression coefficient (SD)  5.08 (1.04), p<0.0001  ICU LOS, regression coefficient (SD)  2.73 (0.90), p=0.0033  Ventilator time, regression coefficient (SD)  2.61 (0.94), p<0.0001  ARDS, OR (95% CI)  NR, NS  Major systemic complication, OR (95% CI)  NR, NS*  Mortality (in hospital), OR (95% CI)  NR, NS  Multivariate analysis time to surgery >72h with reference category <24h  Hospital LOS, regression coefficient (SD)  12.21 (1.47), p<0.0001  ICU LOS, regression coefficient (SD)  7.64 (1.52), p<0.0001  Ventilator time, regression coefficient (SD)  6.52 (0.98), p<0.0001  ARDS, OR (95% CI)  1.75 (1.06-2.87), p<0.0283  Major systemic complication, OR (95% CI)  NR, NS*  Mortality, OR (95% CI)  NR, NS  *Major complications necessitated complex medical intervention such as ARDS, acute kidney injury, pulmonary embolism, unplanned intubation, cardiac arrest requiring cardiopulmonary resuscitation, severe sepsis, ad stroke or cerebrovascular accident | Level of evidence 2009  2b  Risk of bias  Selection bias: –  Performance bias: ?  Attrition bias: +  Detection bias: ?  Authors’ conclusion  “Acute definitive fixation of femoral shaft fractures in the polytraumatically injured patient is safe and is associated with a lower 30-day mortality rate and lower incidence of major systemic complications to include ARDS. Initial management should consist of resuscitation to previously established guidelines before definitive fixation.”  Reviewers’ conclusion  There is a high risk of selection bias as the ISS and GCS differ in the treatment groups. |
| Bläsius 2021  "Strategies for the treatment of femoral fractures in severely injured patients: trends in over two decades from the TraumaRegister DGU." *European Journal of Trauma and Emergency Surgery*. 2021 Feb 15;1-10  Study design  Comparative registry study  (TraumaRegister DGU®)  Aim of the study  To investigate the application frequency of different strategies (ETC, EF and conservative) for the treatment of femoral fractures in severely injured patients over the last two decades. Furthermore, to identify the factors that might influence decision making in choosing one of the aforementioned therapeutic options  Setting  Germany, 2002-2018 | Inclusion criteria   - Age ≥16 years - at least femur fracture and a maximum AIS ≥3 - ISS ≥9 has been previously used - Survival ≥6 h   Exclusion criteria   - Secondary transfer to reporting hospital - Incomplete core dataset (e.g., lack of information about the type of surgery) - Death within 6 hours after admission   Characteristics  Male (%)  IG1: 70.5  IG2: 70.6 CG: 74.3  Age [y], mean ± SD  IG1: 47.0 ± 21.7 IG2: 48.1 ± 22.3 CG: 40.6 ± 18.9  (IG2: vs. CG: p<0.001)  ISS, mean ± SD  IG1: 26.1 ± 15.4 IG2: 19.1 ± 10.7 CG: 25.4 ± 13.4 (IG2: vs. CG: p<0.001)  ISS ≥16, (%)  IG1: 71.3  IG2: 53.5  CG: 73.9  AIS head ≥3, (%)  IG1: 29.4  IG2: 17.0  CG: 27.5  (IG2: vs. CG: p<0.001)  AIS thorax ≥3, (%)  IG1: 46.2  IG2: 31.7  CG: 49.8  (IG2: vs. CG: p<0.001)  AIS abdomen ≥3, (%)  IG1: 14.1  IG2: 9.2  CG: 17.5  (IG2: vs. CG: p<0.001)  Multiple femur fractures, (%)  IG1: 3.7  IG2: 5.1  CG: 14.9  (IG2: vs. CG: p<0.001) | Participants  N=13,091 patients  Study groups  IG1: TC (includes both immobilization in the form of a cast or brace and no immobilization) (N=1601)  IG2: EF (within the DCO concept) (N=5,249)  CG: ETC (N=6,241) | ICU stay: (%)  IG1: 83.8 vs. IG2: 91.9 CG: 96.7  ICU LOS: mean ± SD, day  IG1: 8.2 ± 13.3 vs. IG2: 7.6 ± 11.5 vs. CG: 13.2 ± 15.7  Ventilation time: mean ± SD, day  IG1: 4.2 ± 9.3 vs. IG2: 3.8 ± 9.0 vs. CG: 7.4 ± 12.4  Ventilator-free days: mean ± SD  IG1: 22.0 ± 11.9 vs. IG2: 25.7 ± 8.4 vs. CG: 21.8 ± 10.6  LOS: mean ± SD  IG1: 24.8 ± 25.2 vs. IG2: 23.7 ± 20.4 vs. CG: 32.9 ± 25.9  MOF (%)  IG1: 30.2 vs. IG2: 16.0 vs. CG: 33.1  Sepsis (%)  IG1: 7.4 vs. IG2: 5.4 vs. CG: 11.8  Mortality (in hospital) (%)  IG1: 24.5 vs. IG2: 5.0 vs. CG: 8.7  RISC II prognosis (%)  IG1: 18.8 vs. IG2: 5.9 vs. CG: 10.4  SMR (95% CI)  IG1: 1.30 (1.19–1.41) vs. IG2: 0.85 (0.75–0.95) vs. CG: 0.83 (0.77–0.90) | Level of evidence 2009  2b  Risk of bias  Selection bias: –  Performance bias: ?  Attrition bias: +  Detection bias: ?  Authors´ conclusion  Subgroup analyses revealed that the incidence of sepsis decreased in ETC and EF patients, while the incidence of MOF remained stable in both groups.  Reviewer´s conclusion  There is a high risk of selection bias as the ISS, AIS and the proportion of multiple fractures differ in the treatment groups. |
| Cantu 2014  “In-hospital mortality from femoral shaft fracture depends on the initial delay to fracture fixation and injury severity score- a retrospective cohort study from the NTDB 2002-2006*” J Trauma Acute Care Surg*. 2014; 76(6): 1433–1440  Study design  Comparative registry study  Aim of the study  The purpose of this study was to investigate the optimal time for definitive fixation of femur fractures for patients with varying levels of injury severity.  Setting  USA, 2002-2006 | Inclusion criteria   - Age ≥18 years - Unilateral closed or open femoral shaft fracture (ICD-9 821.01 or 821.11) - Closed or open reduction and internal fixation of the femur (Current Procedural Terminology code 79.15 or 79.35).   Exclusion criteria   - No valid mortality code based on hospital discharge status - No valid ISS code - No valid time to procedure date - No valid admission - transferred from another facility, which could make time to procedure data misleading - transferred out of the facility after surgery, which might bias in-hospital mortality - residing in a burn unit, which might affect surgical timing - missing patient sex designation   Characteristics  NR for subgroup ISS>15 separately | Participants  N=2,323  Study groups  IG: <12h IMN from hospital presentation (N=NR)  IG: 12-24h IMN from hospital presentation (N=NR)  IG: 24-48h IMN from hospital presentation (N=NR)  CG: >48h to 30 days IMN from hospital presentation (N=NR) | Adjusted* in-hospital mortality rates ISS 16-25, % (95% CI)  <12h: 1.53 (0.84–2.77)  12-24h: 2.65 (1.30–5.41)  24-48h: 1.76 (0.59–5.28)  48h-30d: 2.68 (1.05–6.84)  Adjusted* in-hospital mortality rates ISS >25, % (95% CI)  <12h: 5.24 (3.59–7.66)  12-24h: 1.36 (0.36–5.14)  24-48h: 1.26 (0.33–4.83)  48h-30d: 5.90 (3.63–9.60)  *adjusted for full model, surgical timing, injury severity, patient age, sex, race, and Deyo-Charlson comorbidities status  ISS 16-25 time to surgery with reference category <12h  Mortality (in hospital), RR** (95% CI)  >12 to 24h: 1.74 (0.69-4.40)  >24 to 48h: 1.16 (0.33-4.02)  >48h to 30d: 1.76 (0.58-5.33)  ISS 16-25 time to surgery with reference category >12-24h  Mortality (in hospital), RR** (95% CI)  >24 to 48h: 1.50 (0.41-5.55)  >48h to 30d: 1.01 (0.31-3.28)  ISS 16-25 time to surgery with reference category >24-48h  Mortality (in hospital), RR** (95% CI)  >48h to 30d: 1.52 (0.36-6.39)  ISS >26 time to surgery with reference category <12h  Mortality (in hospital), RR** (95% CI)  >12 to 24h: 3.85 (0.97-15.28)  >24 to 48h: 4.16 (1.03-16.75) (significant)  >48h to 30d: 1.12 (0.61-2.08)  ISS >26 time to surgery with reference category >12-24h  Mortality (in hospital), RR** (95% CI)  >24 to 48h: 1.08 (0.16-7.13)  >48h to 30d: 4.33 (1.05-17.79) (significant)  ISS >26 time to surgery with reference category >24-48h  Mortality (in hospital), RR** (95% CI)  >48h to 30d: 4.68 (1.12-19.46) (significant)  **adjusted for sex, race (categorized as white, black, Hispanic, and other), the Deyo-Charlson comorbidity index (dichotomized as 0 and 1+), fracture type (open or closed), and surgical procedure (79.15 closed reduction internal fixation or 79.35 open reduction internal fixation | Level of evidence 2009  2b  Risk of bias  Selection bias: –  Performance bias: ?  Attrition bias: +  Detection bias: ?  Authors’ conclusion  “For patients in the most severely injured ISS group (26+), surgical delay beyond 48 hours was associated with significantly increased mortality risk (ARR, 4.7 vs. within 25–48 hours; 95% CI, 1.1–19.5). The only group in which there was reduced mortality risk with surgical delay was in the most severely injured patients (ISS, 26+) in whom surgical delay of greater than 24 hours but less than 48 hours was associated with the lowest mortality risk compared with surgery within 12 hours (ARR, 4.2; 95% CI, 1.0–16.7)”  Reviewers’ conclusion  Risk of bias in the selection of participants might be controlled due to the division according to ISS. Though it is not clear how ISS is distributed within the ISS divisions. Through the nature of the study a risk of selection bias still exists. |
| Flagstad 2021  “Single-Stage versus Two-Stage Bilateral Intramedullary Nail Fixation in Patients with Bilateral Femur Fractures, A Multicenter Retrospective Review” *J Orthop Trauma* 2021 Jan 5 online ahead of print  Study design  Comparative registry study  Aim of the study  The purpose of our study is to evaluate and compare the rates of complications in patients with bilateral femoral shaft fractures treated with bilateral IMN in either one, single procedure or two, separate procedures.  Setting  NR, 1998-2018 | Inclusion criteria   - Skeletally mature - Bilateral, extra-articular femur fractures - Both fractures treated definitely with IMN   Exclusion criteria   - Intra-articular femur fractures - Died before definitive fixation of both femurs - Definitely treated with plate or external fixation   Characteristics  Age [y], mean ± SD  IG 36.7 ± 14.6 vs. CG: 32.9 ± 15.6, p=0.11  Male, n (%)  IG 34 (58.6) vs. CG: 123 (65.4), p=0.35  ISS, mean ± SD  IG 24.9 ± 11.9 vs. CG: 24.5 ± 11.9, p=0.79  GCS, mean ± SD  IG 11.4 ± 5.1 CG: 12.3 ± 4.5, p=0.22  Open fracture n, (%)  Unilateral:  IG: 13 (22.8) vs. CG: 52 (29.5)  Bilateral:  IG: 3 (5.3) vs. CG: 12 (6.6.), p=0.54  Secondary Injuries, n (%)  Head:  IG: 22 (37.9) vs. CG: 66 (35.3), p=0.711  Chest:  IG: 31 (53.4) vs. CG: 86 (46.0), p=0.321  Abdominal:  IG: 21 (36.2) vs. CG: 79 (42.0), p=0.431  AIS, mean ± SD  Head:  IG: 1.2 ± 1.5 vs. CG: 1.3 ± 1.6, p=0.45  Face:  IG: 0.5 ± 0.8 vs. CG: 0.5 ± 0.8, p=0.832  Neck:  IG: 0.3 ± 0.9 vs. CG: 0.2 ± 0.7, p=0.553  Chest:  IG: 2.0 ± 1.7 vs. CG: 1.8 ± 1.7, p=0.432  Abdomen:  IG: 1.3 ± 1.6 vs. CG: 1.1 ± 1.4, p=0.472  Spine:  IG: 0.6 ± 0.9 vs. CG: 0.5 ± 1.0, p=0.692  Extremity:  IG: 3.2 ± 0.5 vs. CG: 3.2 ± 0.4, p=0.613  Time to definitive fixation hours, mean ± SD  IG: 163.5 ± 218.4 vs. CG: 52.6 ± 140.3, p<0.013  Time to 1st IMN fixation hours, mean ± SD IG: 86.4 ± 218.4 vs. CG: N/A  Time between 1st and 2nd IMN fixation hours, mean ± SD  IG: 103.2 (180) vs. CG: N/A  Placement of external fixator, n (%)  IG: 45 (78) vs. CG: 31 (16), p<0.011  Definitive Fixation ≤24h, n (%)  IG: 1 (1.7) vs. CG: 132 (70), p<0.011  Definitive Fixation >24-72h, n (%)  IG: 17 (29.3) vs. CG: 32 (17), p=0.041  Definitive Fixation >72-120h, n (%)  IG: 13 (22) vs. CG: 8 (4), p<0.011  Definitive Fixation >120h, n (%)  IG: 16 (9) vs. CG: 27 (47), p<0.011 | Participants  N=246 patients  Study groups  IG: IMN during two separate procedures (two-stage) (N=58)  CG: IMN during one procedure (single-stage) (N=188) | Hospital LOS days: mean ± SD  IG 28.5 ± 29.5 vs. CG: 16.4 ± 28.5, p<0.012  ICU LOS days: mean ± SD  IG 11.8 ± 12.8 vs. CG: 7.6 ± 8.2, p=0.023  Unadjusted outcomes:  Mortality (in hospital): n (%)  IG 0 (0.0) vs. CG: 5 (2.7), p=0.22  Rhabdomyolysis: n (%)  IG 7 (12.1) vs. CG: 0 (0.0%), p<0.01  60-Day Readmission: n (%)  IG 7 (12.3) vs. CG: 18 (9.6), p=0.56  Pulmonary Complications*: n (%)  IG 26 (44.8) vs. CG: 46 (24.5)  Fat Emboli Syndrome: n (%)  IG 6 (10.3) vs. CG: 11 (5.9), p=0.24  Pulmonary Embolism: n (%)  IG 4 (6.9) vs. CG: 7 (3.7), p=0.31  ARDS: n (%)  IG 8 (13.8) vs. CG: 11 (5.9), p=0.05  Hospital-Acquired Pneumonia: n (%)  IG 3 (5.2) vs. CG: 4 (2.1), p=0.22  Ventilator-Acquired Pneumonia: n (%)  IG 5 (8.6) vs. CG: 13 (7.0), p=0.67  Deep Vein Thrombosis: n (%)  IG 6 (10.3) vs. CG: 10 (5.4), p=0.18  Acute Stroke: n (%)  IG 2 (3.4) vs. CG: 4 (2.1), p=0.59  Inpatient Dialysis: n (%)  IG 5 (8.6) vs. CG: 2 (1.1), p<0.01  Sepsis: n (%)  IG 4 (7.0) vs. CG: 9 (4.9), p=0.53  Adjusted* outcomes reporting RRs for single-stage procedure, RR (95% CI)  Fat Emboli Syndrome: 0.50 (0.15-1.63), p=0.25  Pulmonary Embolism: 1.56 (0.27-8.14), p=0.62  ARDS: 0.22 (0.06-0.75), p=0.02  Hospital-Acquired Pneumonia: 0.06 (0.01-1.53), p=0.09  Ventilator-Associated Pneumonia: 0.59 (0.17-2.08), p=0.41  Deep Vein Thrombosis: 0.67 (0.16-2.73), p=0.58  Acute Stroke: 0.43 (0.05-3.44), p=0.42  Inpatient Dialysis: 0.16 (0.01-2.07), p=0.16  Sepsis: 0.44 (0.12-1.64), p=0.22  *Adjusted for age, gender, ISS, AIS, GCS, and admission lactate | Level of evidence 2009  2b  Risk of bias  Selection bias: –  Performance bias: ?  Attrition bias: +  Detection bias: +  Authors’ conclusions  Polytrauma patients may benefit from single-stage bilateral femur IMN if able to receive definitive fixation no later than 48 hours from admission. However, a larger study is required to discern whether single- versus two-stage fixation has an effect on mortality and to identify the individuals at risk for mortality.  Reviewers’ conclusion  There is a high risk of selection bias in the study as the two-stage group underwent IMN (first procedure and definitive procedure) later than the single-stage group |
| Morshed 2009  “Delayed Internal Fixation of Femoral Shaft Fracture Reduces Mortality Among Patients with Multisystem Trauma” *J Bone Joint Surg Am*. 2009;91:3-13  Study design  Comparative registry study  Aim of the study  The effect of the timing of definitive care of femoral shaft fractures with use of a multilevel definition of treatment time drawn from the largest available multicenter database of patients with multisystem trauma was studied (the National Trauma Data Bank).  Setting  USA 2000-2004 | Inclusion criteria   - Age ≥16 years - closed or open fracture (or fractures) of the femoral shaft (ICD-9 821.0/ 821.01/821.1/821.11) - ISS ≥15 - definitive treatment procedure involving internal fixation of the - femur as identified by an ICD-9-CM procedure code of 78.55 (internal fixation—femur), 79.15 (closed reduction and internal fixation—femur), or 79.35 (open reduction and internal fixation—femur)   Exclusion criteria   - patient was received in transfer or was not admitted on the day of injury - record lacked information on time from admission to definitive fracture fixation, mortality status, or length of hospitalization, or on time of surgery - associated burn - fracture was not definitely fixed within two weeks of admission   Characteristics  Age [y], mean ± SD  IG1: 31.86 ± 13.73 IG2: 33.12 ± 15.73 IG3: 34.25 ± 15.87 IG4: 34.44 ± 16.33 CG: 33.38 ± 15.05), p=0.01  NISS, mean ± SD  IG1: 27.35 ± 8.97 IG2: 27.21 ± 8.33 IG3: 29.20 ± 9.62 IG4: 32.31 ± 11.39 CG: 34.68 ± 14.05), p<0.001  GCS, mean ± SD  IG1: 12.67 ± 4.22 IG2: 12.69 ± 4.14 IG3: 11.66 ± 4.75 IG4: 10.77 ± 5.13 CG: 9.68 ± 5.20), p<0.001  Maximum AIS head/neck region, mean ± SD  IG1: 1.71 ± 1.65 IG2: 1.76 ± 1.69 IG3: 2.01 ± 1.68  IG4: 2.32 ± 1.83 CG: 2.50 ± 1.94), p<0.001  Number of serious associated extremity injuries, mean ± SD  IG1: 1.65 ± 0.94 IG2: 1.42 ± 0.73 IG3: 1.54 ± 0.86 IG4: 1.67 ± 1.01 CG: 1.68 ± 0.96), p<0.001  Bilateral fracture, proportion (SD)  IG1: 0.02 (0.14)  IG2: 0.00 (0.06)  IG3: 0.01 (0.12)  IG4: 0.02 (0.12)  CG: 0.02 (0.15), p=0.03 | Participants  N=3,069 patients  Study groups  IG: <12h definitive fixation (N=1,759)  IG: 12-24h definitive fixation (N=540)  IG: 24-48h definitive fixation (N=359)  IG: 48-120h definitive fixation (N=272)  CG: >120h definitive fixation (N=139) | Adjusted* in-hospital mortality reporting RRs time to surgery with reference category <12h, inverse probability of treatment-weighted RR (95% CI)  12 to 24h: 0.45 (0.15-0.98), p=0.03  24 to 48h: 0.83 (0.43-1.44), p=0.49  48 to 120h: 0.58 (0.28-0.93), p=0.03  >120h: 0.43 (0.10-0.94), p=0.03  *Adjusted for NISS, GCS, Northeast region, age, arrival time, the number of serious extremity/pelvic or head/neck injuries, the number of femoral fractures, the presence of cardiac or cerebrovascular comorbidities, teaching status, and American College of Surgeons level-1 designation  Standardized risk ratio**  12 to 24h: 0.47 (0.14 to 1.11), p=0.07  24 to 48h: 0.94 (0.44 to 1.76), p=0.85  48 to 120h: 0.58 (0.21 to 1.09), p=0.09  >120h: 0.43 (0.09 to 0.94), p=0.05  **The standardized risk ratio analysis involved the use of the same treatment model as inverse probability of treatment-weighted analysis but modified weights to give the estimated proportionate risk that would have been observed if the subjects in the early treatment group (<12h) had received treatment at a later time. | Level of evidence 2009  2b  Risk of bias  Selection bias: –  Performance bias: ?  Attrition bias: +  Detection bias: ?  Authors’ conclusion  “We estimate an approximate 50% reduction in the risk of mortality when treatment between twelve and twenty-four hours and more than forty-eight hours is compared with treatment within twelve hours after admission; a nonsignificant reduction is estimated when treatment occurs between twenty-four and forty-eight hours.. These results provide strong empirical evidence in support of a delayed or ‘‘damage-control’’ approach to definitive fixation of femoral shaft fracture among patients with multisystem trauma.”  Reviewers’ conclusion  There is a high risk of selection bias as the group 12-24h has the lowest NISS, GCS and AIS compared to the other groups. |
| Morshed 2015  “Timing of Femoral Shaft Fracture Fixation Affects Length of Hospital Stay in Patients with Multiple Injuries” *The Open Orthopaedics Journal*, 2015, 9, (Suppl 1: M8) 324-331  Study design  Comparative registry study  Aim of the study  The current study addresses the impact of timing of definitive fixation on the length of hospital stay with use of a multilevel definition of treatment time drawn from the largest available multi-center database of patients with multisystem trauma (the National Trauma Data Bank).  Setting  USA 2000-2004 | Inclusion criteria   - Age ≥16 years - closed or open fracture (or fractures) of the femoral shaft (ICD-9 821.0/ 821.01/821.1/821.11) - ISS ≥15 - definitive treatment procedure involving internal fixation of the - femur as identified by an ICD-9-CM procedure code of 78.55 (internal fixation—femur), 79.15 (closed reduction and internal fixation—femur), or 79.35 (open reduction and internal fixation—femur)   Exclusion criteria   - patient was received in transfer or was not admitted on the day of injury - record lacked information on time from admission to definitive fracture fixation,mortality status, or length of hospitalizationfracture was not definitely fixed within two weeks of admission - the patient had a negative post-operative LOS   Characteristics  Age [y], mean ± SD  IG1: 31.9 ± 13.7 IG2: 32.7 ± 15.8 IG3: 34.2 ± 15.9 IG4 34.5 ± 16.4 CG: 33.4 ± 15.1, p=0.01  NISS, mean ± SD  IG1: 27.3 ± 8.95 IG2: 27.2 ± 8.43 IG3: 29.2 ± 9.68 IG4 32.4 ± 11.52 CG: 34.6 ± 14.03, p<0.001  GCS, mean ± SD  IG1: 12.68 ± 4.21 IG2: 12.70 ± 4.11 IG3: 11.86 ± 4.61 IG4 10.86 ± 5.08 CG: 9.83 ± 5.16, p<0.00)  Maximum AIS head/neck region, mean ± SD  IG1: 1.71 ± 1.65 IG2: 1.76 ± 1.69 IG3: 2.01 ± 1.68 IG4 2.32 ± 1.83 CG: 2.50 ± 1.94, p<0.001  Number of serious associated  extremity injuries, mean ± SD  IG1: 1.64 ± 0.94 IG2: 1.39 ± 0.71 IG3: 1.50 ± 0.79 IG4 1.67 ± 1.01 CG: 1.68 ± 0.96, p<0.001  Bilateral fracture, proportion (SD)  IG1: 0.02 (0.14)  IG2: 0.00 (0.06)  IG3: 0.01 (0.12)  IG4 0.02 (0.12)  CG: 0.02 (0.15), p=0.03 | Participants  N=2,949 patients  Study groups  IG: <12h definitive fixation (N=1,685)  IG: 12-24h definitive fixation (N=518)  IG: 24-48h definitive fixation (N=347)  IG: 48-120h definitive fixation (N=263)  CG: >120h definitive fixation (N=136) | Adjusted* post-operative hospitalization for time to surgery with reference category <12h, days median difference (95% CI)  12 to 24h: -0.61 (-1.53, 0.42), p=0.2949  24 to 48h: -0.00 (-1.00, 1.47), p=0.9984  48 to 120h: 2.77 (0.54, 4.72), p=0.0080  >120h: 0.86 (-2.00, 4.15), p=0.7351  Adjusted* post-operative hospitalization for time to surgery with reference category <12h deaths excluded, days median difference (95% CI)  12 to 24h: -0.64 (-1.53, 0.3.8), p=0.2949  24 to 48h: 0.24 (-0.96, 1.61), p=0.6346  48 to 120h: 2.53 (0.27, 4.13), p=0.0167  >120h: 0.55 (-2.04, 4.08), p=0.7674  Adjusted* post-operative hospitalization for time to surgery with reference category <12h death imputed to 220 days, days median difference (95% CI)  12 to 24h: -1.18 (-1.53, 0.38), p=0.1261  24 to 48h: 0.33 (-0.91, 2.00), p=0.6708  48 to 120h: 2.28 (0.28, 4.42), p=0.0317  >120h: 0.18 (-2.63, 4.00), p=0.8899  *Adjusted for hospital arrival time, NISS, cardiovascular disease, cerebrovascular disease, number of severe (AIS >3) extremity injuries, age, maximum head region AIS, teaching status of treating facility, GCS, trauma center designation. Increased probability treatment weighted. | Level of evidence 2009  2b  Risk of bias  Selection bias: –  Performance bias: ?  Attrition bias: +  Detection bias: ?  Authors’ conclusion  “We estimate a 2 to 3 day increase in the median LOS when treatment occurs between forty-eight and 120 hours after admission; a non-significant reduction is estimated when treatment occurs between twelve and twenty-four hours. These results provide strong empirical evidence in support of a delayed or “damage control” approach to definitive fixation of femoral shaft fracture among multisystem trauma patients. Our findings suggest that patients who are not adequately resuscitated by their 2nd hospital day should likely not undergo definitive femoral fixation until the fifth hospital day as this period corresponds to peaking of systemic inflammatory response and susceptibility to “second hit” from a major surgical intervention.”  Reviewers’ conclusion  There is a high risk of selection bias as the group 12-24h has the lowest NISS and AIS compared to the other groups. Furthermore, it should be noted, that AIS score and the proportion of bilateral fractures are exactly the same as in Morshed et al. 2009 (see above) even though the population differs slightly. |
| Richards 2020  “Musculoskeletal Trauma in Critically Injured Patients: Factors Leading to Delayed Operative Fixation and Multiple Organ Failure*“ Anesthesia & Analgesia*: December 2020 - Volume 131 - Issue 6 - p 1781-1788  Study design  Comparative registry study  Aim of the study  The purpose of this investigation was 2-fold: (1) Describe clinical variables that were associated with late definitive fixation in a cohort of critically injured trauma patients with femur fractures and (2) explore the association between late definitive fixation and MOF.  Setting  NR, 2009-2016 | Inclusion criteria   - age 18–89 - ISS >15 - Femur fracture repaired definitively with internal fixation - Admission to the ICU directly from the scene of injury   Exclusion criteria   - Head AIS >3 - Fewer than 2 lactate levels within 24 h of admission - ICU LOS <2 d - Femur fracture treated nonoperatively - Pathologic fractures   Characteristics  Age, median (IQR)  IG: 33.4 (24.1–47.3) vs. CG: 34.0 (24.0–49.0), p=0.88  ISS, mean ± SD  IG: 26.0 ± 6.9 vs. CG: 30.9 ± 9.4, p<0.001  Head AIS, median (IQR)  IG: 0 (0–3) vs. CG: 1 (0–3), p=0.36  Chest AIS, median (IQR)  IG: 3 (2–4) vs. CG: 3 (3–4), p=0.11  Abdominal AIS, median (IQR)  IG 2 (0–2) vs. CG: 2 (0–3), p=0.0001  ED GCS, mean ± SD  IG: 11.4 ± 5.1 vs. CG: 11.4 ± 5.0, p=0 .9 | Participants  N=279 patients  Study groups  IG: early fixation (within 24 hours of hospital admission) (N=160)  CG: late fixation (≥24 hours after Admission) with temporary fixation with spanning external fixation or skeletal traction (N=119) | MOF within 28 days of injury % (95% CI)  IG 11.0 (2.7-18.5) vs. CG: 42.2 (25.3-55.2) difference 31.2 (14.4-48.0), p<0.001  After adjusting for observed confounding using inverse probability weighting late fixation was associated with a 3-fold increase in the risk of MOF  (HR = 3.21, 95% CI, 1.48-7.00; p<0.01) | Level of evidence 2009  2b  Risk of bias  Selection bias: –  Performance bias: ?  Attrition bias: +  Detection bias: ?  Authors’ conclusion  “Higher ISS and a greater depth of shock during the initial 24 hours of hospitalization were associated with late fracture fixation. Furthermore, late femur fracture fixation was significantly associated with 28-day MOF.”  Reviewers’ conclusion  There is a high risk of selection bias in the study as the late fixation group is injured more severely. Furthermore the authors report that they not reached the planned numbers. Which might be a result of attrition bias as more patients were excluded from the study than anticipated. |
| Rixen 2016  "Randomized, controlled, two-arm, interventional, multicenter study on risk-adapted damage control orthopedic surgery of femur shaft fractures in multipletrauma patients." Trials (2016) 17:47.  Study design  Randomised controlled trial  Aim of the study  This study investigates whether the use of damage control through the application of external fixation to the femoral shaft fractures in severely injured multiple trauma patients will reduce the risk of mortality as measured by the sepsis-related organ failure assessment score when compared to early IMN.  Setting  Germany, 2007-2009 | Inclusion criteria   - age ≥18 years - multiple trauma (injury of at least two body regions) with an ISS ≥16 - a femoral shaft fracture which can be treated in principle by nail or fixateur externe (surgical treatment beginning within 24 hours after trauma) - calculated probability of death between 20-60%   Exclusion criteria   - III° open fractures - refusal of one of both strategies by either the investigator or the patient, - start of internal or external fracture fixation before randomization - participation in concurrent interventional studies - pregnancy   Characteristics  Age [y], mean ± SD  IG: 39.4 ± 15.3 vs. CG: 38.9 ± 15.3  Male, n (%)  IG: 12 (70.5) vs. CG: 12 (75)  ISS, mean ± SD  IG: 39.8 ± 8.9 vs. CG: 41.4 ± 15.7  GCS, mean ± SD  IG 7.0 ± 3.6 CG: 8.5 ± 3.2  Calculated probability of death % (SD)  IG: 31 (13) vs. CG: 30 (12) | Participants  N=30 patients  Study groups  IG: EF and secondary reamed IMN (N=17, N=16 received the allocated treatment)  Secondary surgery could be performed as soon as the patients treated with external fixation were stabilized with ventilation (paO2/FiO2 >200 if ventilated or no need for ventilation), coagulation (prothrombin time >60% and platelets >60,000/μl), hemodynamics (no need for noradrenalin or adrenalin and mean arterial pressure >60 mmHg), the metabolic system (BE >-6.0 mmol/l), and furthermore showed no  signs of systemic or local inflammation.  CG: primary reamed nailing N=17 randomized but 1 died after randomization but before treatment (N=14 received the allocated treatment) | Maximal sepsis-related organ failure assessment score within 28 days after trauma mean ± SD  IG 8.7 ± 3.8 vs. CG: 9.6 ± 5.1, p=0.510  Cumulative sepsis-related organ failure assessment score within 28 days after trauma mean ± SD  IG 112.4 ± 118.8 vs. CG: 113.8 ± 166.6, p=0.254  Transfusion requirements during surgery (packed red blood cells) mean ± SD  IG 4.7 ± 4.8 vs. CG: 6.6 ± 6.1, p=0.350  ICU LOS (days) mean ± SD  IG 21.8 ± 13.9 vs. CG: 12.38 ± 9.9, p=0.037  Days of ventilation mean ± SD  IG 15.0 ± 9.6 vs. CG: 8.6 ± 7.9, p=0.049  In-hospital LOS (days) mean ± SD  IG 32.3 ± 20.2 vs. CG: 30.2 ± 18.2, p=1.0 | Level of evidence 2009  1b  Risk of bias  Selection bias: +  Performance bias: ?  Attrition bias: +  Detection bias: +  Authors’ conclusion  “In conclusion, the results of this randomized study reflect the ambivalence in the literature. In correspondence to the systematic review [4], we could not find advantages of the damage control concept in the treatment of femoral shaft fractures in the care of multiple trauma patients. Unfortunately, our results are not statistically significant due to the small number of included patients.”  Reviewers’ conclusion  The study seems to have a low risk of bias due to randomization and with that comparable treatment groups regarding injury severity. |
| Steinhausen 2014  “A risk-adapted approach is beneficial in the management of bilateral femoral shaft fractures in multiple trauma patients: An analysis based on the trauma registry of the German Trauma Society”. *J Trauma Acute Care Surg* 2014; 76(5): 1288-93.  Study design  Comparative registry study  Aim of the study  This study aimed to validate that a risk-adapted approach in the management of multiple trauma patients with bilateral femoral shaft fractures results in low mortality and acute morbidity on the basis of the trauma registry of the German Trauma Society.  Setting  Germany, 1993-2008 | Inclusion criteria   - All multiple trauma patients with bilateral femur shaft fracture were included.   Characteristics  ISS, mean ± SD  IG1: 31.1 ± 15.0 IG2: 23.5 ± 12.0 IG3: 19.9 ± 10.9 CG: 35.8 ± 18.5 (IG1: vs. IG2: p<0.001)  Thoracic AIS score ≥3, %  IG1: 65.3  IG2: 43.2  IG3: 37.3  CG: 68.8 (IG1: vs. IG2: p=0.001)  Head AIS score ≥3, %  IG1: 40.4  IG2: 30.5  IG3: 16.9  CG: 43.8 (IG1: vs. IG2: p=0.12)  Abdominal AIS score ≥3, %  IG1: 23.8  IG2: 15.8  IG3: 18.6  CG: 34.4 (IG1: vs. IG2: p=0.13) | Participants  N=379 patients  Study groups  IG1: DCO with bilateral EF (N=193)  IG2: ETC with bilateral primary definitive osteosynthesis (N=95)  IG3: ETC of one FSF and DCO of the contralateral side (mixed) (N=59)  CG: no osteosynthesis (N=32) | Hospital mortality: n (%)  IG1: 26 (13.5) vs. IG2: 8 (8.4) vs. IG3: 1 (1.7) vs. CG: 21 (65.6)  ICU stay, days: mean ± SD  IG1: 19.4 ± 25.6 vs. IG2: 11.5 ± 9.9 vs. IG3: 13.3 ± 15.1 vs. CG: 6.2 ± 7.3 (IG1: vs. IG2: p<0.001)  Ventilation time, days: mean ± SD  IG1: 11.6 ± 15.6 vs. IG2: 7.3 ± 9.4 vs. IG3: 11.3 ± 14.9 vs. CG: 3.5 ± 4.5 (IG1: vs. IG2: p=0.005)  Ventilator-free days: mean ± SD  IG1: 17.5 ± 10.8 vs. IG2: 21.2 ± 9.7 vs. IG3: 20.1 ± 9.7 vs. CG: 6.6 ± 10.9 (IG1: vs. IG2: p=0.008)  OF: %  IG1: 55.3 vs. IG2: 39.6 vs. IG3: 37.5 vs. CG: 40.9 (IG1: vs. IG2: p=0.020)  MOF: %  IG1: 40.2 IG2: 25.3 IG3: 23.2 CG: 22.7 (IG1: vs. IG2: p=0.016)  Sepsis: %  IG1: 21.6 vs. IG2: 12.0 IG3: 13.5 vs. CG: 0.0 (IG1: vs. IG2: p=0.081) | Level of evidence 2009  2b  Risk of bias  Selection bias: –  Performance bias: ?  Attrition bias: +  Detection bias: ?  Authors’ conclusion  “a risk-adapted approach to bilateral femoral shaft fractures resulted in low mortality and acute morbidity in our study. The clearly stable patient was reasonably treated with IMN. The unstable or potentially unstable patient was reasonably treated with DCO. An increased ISS and in particular the presence of severe thoracic injury as well as coagulopathy stratified the patients at risk and influenced decision making. When in doubt, the patient is probably not totally stable, and the safest precaution is to use DCO as a risk adapted approach.”  Reviewers’ conclusion  There is a high risk of selection bias, as the group with DCO is more severely injured than that with ETC or mixed, while no osteosynthesis has the most severe injuries. GCS was supposed to be reported but does not appear in the publication. Furthermore missing (or incomplete) data limits the analysis (patients not undergoing any operation) |
| Stojiljković 2009  “Damage control strategy in the treatment of closed femoral shaft fractures in polytrauma patients” *ACTA FAC MED NAISS* 2009; 26 (3): 127-133  Study design  Prospective cohort study  Aim of the study  The aim of the paper was to present the advantages of the 'damage control' strategy in the management of closed femoral shaft fractures in polytrauma patients.  Setting  Serbia, 1999-2006 | Inclusion criteria   - Polytrauma patients - With closed femoral fractures   Characteristics  Age [y], mean (range)  34.27 (16-67)  Male gender, n (%)  48 (70.59)  Trauma Score, n (%)  Stable (13-16): 13 (19.12)  Borderline (9-12): 26 (38.23)  Unstable and critical (1-8): 29 (42.65)  Bilateral femur fracture, n (%)  2(2.94)  Other injuries, n (%)  Head injuries: 29 (42.65)  Abdominal injuries: 11 (16.17)  injuries of the locomotor system: 16 (23.53)  Chest injuries 12 (17.65)  Conversion of externalinto internal fixation mean, days (range)  21.6 (21-40) | Participants  N=70 patients  Study groups  IG1: external fixation (via Mitković external fixator) (N=9)  IG2: external fixation converting to internal fixation (N=5)  CG: internal fixation (Kuntsher nail (intramedullary fixation) and self-dynamisable internal fixator by Mitkovic were used) (N=56)  The fractures were temporarily managed by the skeletal traction and coxofemoral plaster cast immobilization until the surgical treatment of femoral fracture. | Healing time months median  IG1: (SD) 6.11 (0.81) vs. CG: NR, but significant longer (p<0.005)  Functional assessment bad: n (%)  IG1: 1 (11.1) vs. IG2: 0 (0), vs. CG: 3 (5.4)  Functional assessment poor: n (%)  IG1: 1 (11.1) vs. IG2: 0 (0), vs. CG: 0 (0)  Functional assessment good: n (%)  IG1: 5 (55.6) vs. IG2: 2 (40), vs. CG: 15 (26.8)  Functional assessment excellent: n (%)  IG1: 2 (22.2) vs. IG2: 3 (60), vs. CG: 38 (67.9)  Pin track infection: n (%)  IG1: 2 (22.2) vs. IG2: 0 (0), vs. CG: 0 (0)  Deep pin track infection: n (%)  IG1: 1 (11.1) vs. IG2: 0 (0), vs. CG: 0 (0)  Early disintegration of Implants: n (%)  IG1: 0 (0) vs. IG2: 0 (0), vs. CG: 1 (1.78)  Nonuion: n (%)  IG1: 1 (11.1) vs. IG2: 0 (0), vs. CG: 2 (3.58)  Osteitis: n (%)  IG1: 0 (0) vs. IG2: 0 (0), vs. CG: 1 (1.78)  The presence or absence of the local complications in regard to the femoral fracture management showed statistically significant difference (x =9.34 p<0.01), i.e. there were more complications which followed external skeletal fixation as the definitive treatment method.  There was statistically significant difference in blood substitution during the intervention (x =18.60 p<0.005) and the postoperative blood loss through  the operative wound drainage after orthopaedic intervention (x =77.53 p<0.001). Blood loss during external skeletal fixation (operative and postoperative) in our series was statistically significantly smaller than in the applied methods of internal fixation. | Level of evidence 2009  2b  Risk of bias  Selection bias: –  Performance bias: ?  Attrition bias: +  Detection bias: +  Authors’ conclusion  “The obtained results confirm the hypothesis that early internal fixation of the femoral fractures in the polytrauma patients poses great and additional trauma for the injured, and it can be safely performed after stabilizing the patient condition. External fixation stands for a safe operative method for accomplishing temporary stability of the femoral fracture in the polytraumatized patients and a minimally additional operative trauma. However, it is associated with a number of complications and a worse functional outcome when compared to the internal fixation method. External fixation of the femoral fracture in the polytraumatized patients should be converted into internal fixation when the patient's condition allows.”  Reviewers’ conclusion  There is a risk of selection bias due to the nature of the study. Patient characteristics in the study groups remain unknown. |

##### Recommendation 3.82

| Study: Reference, aim, design, setting | Participants: selection criteria, characteristics | N Participants; Intervention (IG) vs. Control group (CG) | Main outcomes | Assessment: LoE, risk of bias; Conclusions |
| --- | --- | --- | --- | --- |
| Doukas (2013)  “The Military Extremity Trauma Amputation/Limb Salvage (METALS) Study*” J Bone Joint Surg Am.* 2013;95:138-45  Study design  Comparative registry study  Aim of the study  The objective of this study was to examine functional outcomes and disability following major lower-extremity trauma sustained in the military and to compare the outcomes between patients treated with amputation and those treated with limb salvage.  Setting  US military (Afghanistan/Iraq), 2003-2007 | Inclusion criteria  active duty personnel and reservists deployed to Afghanistan or Iraq who had sustained an injury to the upper or lower limb (excluding the pelvis/acetabulum) that resulted in a major amputation (at or proximal to the hindfoot or the radiocarpal joint) or required operative treatment and revascularization, bone-grafting/bone transport, local/ free flap coverage, repair of a major nerve injury, or treatment of a complete compartment injury/compartment syndrome  Exclusion criteria  GCS <15 at discharge or spinal cord injury  Characteristics  Unilateral Lower-Limb Injury  Male gender (%)  IG 98.2 vs. CG: 97.0  Age (%)  18-24 years: IG 26.5 vs. CG: 24.0  25-29 years: IG 36.3 vs. 35.2  ≥30 years: IG 37.2 vs. CG: 40.8  Time to interview, month mean  IG 37.4 vs. CG: 39.5  Bilateral Lower-Limb Injury  Male gender (%)  98.8  Age (%)  18-24 years: 21.2  25-29 years: 37.6  ≥30 years: 41.2  Time to interview, month mean  34.8 | Participants  N=324 patients  Study groups  *Unilateral Lower-Limb Injury*  IG: Amputation (N=113)  CG: Salvage (N=126)  *Bilateral Lower-Limb Injury*  IG1: Bilateral Amputation (N=39)  IG2: Amputation and Salvage (N=30)  CG: Bilateral Salvage (N=16) | Unilateral Lower-Limb Injury  Adjusted* SMFA scores, mean  Total dysfunction: IG 21.5 vs. CG: 29.8, p<0.01  Mobility: IG 27.5 vs. CG: 37.2, p<0.01  Daily activities: IG 20.4 vs. CG: 27.9, p<0.05  Emotional status: IG 37.6 vs. CG: 47.9, p<0.01  Arm/hand function: IG 2.1 vs. CG: 8.2, p<0.01  Engaged in vigorous sports or recreational activities (%)  IG 45.1 vs. CG: 26.2  With depressive symptoms (%)  IG 40.7 vs. CG: 43.6  With possible/probable major depression (%)  IG 13.3 vs. CG: 15.1  Screened positive for PTSD (%)  IG 14.8 vs. CG: 26.8  Working/on active duty (%)  IG 43.4 vs. CG: 48.0  In school (%)  IG 29.2 vs. CG: 18.4  With pain interfering with daily activity (%)  IG 17.1 vs. CG: 27.0  Bilateral Lower-Limb Injury  Adjusted* SMFA scores, mean  Total dysfunction: IG1: 22.2 vs. IG2: 24.0‡ vs. CG: 30.0  Mobility: IG1: 30.2 vs. IG2: 30.8‡ vs. CG: 40.3  Daily activities: IG1: 22.8 IG2: 27.5 CG: 26.8  Emotional status: IG1: 33.2‡ vs. IG2: 32.0† vs. CG: 44.4  Arm/hand function: IG1: 3.0‡ vs. IG2: 5.1 vs. CG: 10.0  Engaged in vigorous sports or recreational activities (%)  IG1: 48.7 vs. IG2: 50.0 vs. CG: 31.2  With depressive symptoms (%)  IG1: 25.6 vs. IG2: 23.3 vs. CG: 37.5  With possible/probable major depression (%)  IG1: 10.3 vs. IG2: 6.7 vs. CG: 12.5  Screened positive for PTSD (%)  IG1: 10.3 vs. IG2: 6.4 vs. CG: 12.5  Working/on active duty (%)  IG1: 30.8 vs. IG2: 36.7 vs. CG: 56.2  In school (%)  IG1: 17.9 vs. IG2: 6.6 vs. CG: 6.3  With pain interfering with daily activity (%)  IG1: 10.3 vs. IG2: 16.7 vs. CG: 12.5  *Adjusted for the presence of Military Extremity Trauma Amputation/Limb Salvage-eligible upper-limb injury, presence of bilateral lower-limb injury, months until interview, age, military rank, intensity of combat experiences, presence of social support. | Level of evidence 2009  2b  Risk of bias  Selection bias: –  Performance bias: ?  Attrition bias: –  Detection bias: ?  Authors’ conclusion  “At an average of three years postinjury, those treated with amputation appeared to have better functional outcomes than those treated with limb salvage. Prospective studies are needed to confirm these results and to determine the role that rehabilitation protocols, ancillary services, or other external factors play in determining better or worse outcomes. At the present time, data are insufficient to support the selection of amputation over limb salvage. Rather, our results underscore the importance of addressing the post-acute-care needs of both patients treated with limb salvage and those who undergo amputation.”  Reviewers’ conclusion  It is unclear whether patients with amputations or salvage were injured more severely. Moreover, there seem to be a high risk of bias as response was voluntary and potentially only those with a very good or very bad outcome took part in the survey. |
| AIS Abbreviated injury scale, ARDS Acute respiratory distress syndrome, ARR adjusted relative risk, BE base excess, CG: control group, CI confidence interval, DCO Damage control orthopaedics, DGU Deutsche gesellschaft für Unfallchirurgie, ED emergency department, EF Temporary external fixation, ETC Early TOTAL Care, GCS Glasgow coma scale, ICU Intensive care unit, IG intervention group, IMC Intermediate care, IMN intramedullary nailing, ISS Injury severity score, LOS length of stay, MAIS Maximum abbreviated injury scale, MOF Multiple organ failure, MV Mechanical ventilation, NISS New Injury Severity Score, NR not reported, NS not significant, OR Odds ratio, PTSD posttraumatic stress disorder, RISC II Revised injury severity score II, RR relative risk, SD Standard deviation, SDS Safe definitive surgery, SMR Standardized mortality ratio, SMFA Short Musculoskeletal Function Assessment, TC Conservative treatment | | | | |

# S5 Deleted Recommendations

| 3.67 | Isolierte und multiple Schaftfrakturen langer Röhrenknochen der unteren Extremität können beim Polytrauma des Erwachsenen sowohl primär-definitiv als auch primär-temporär und se-kundär-definitiv osteosynthetisch versorgt werden.  *2011* | 0 |
| --- | --- | --- |
| 3.68 | Isolierte geschlossene Schaftfrakturen der Tibia können ausnahmsweise auch im Gipsverband primär-temporär stabilisiert werden.  *2011* | 0 |
| 3.69 | Proximale Femurfrakturen beim Polytrauma können primär osteosynthetisch stabilisiert werden.  *2011* | 0 |
| 3.70 | In begründeten Fällen kann vorübergehend ein gelenkübergreifender Fixateur externe indiziert sein.  *2011* | 0 |
| 3.72 | Instabile distale Femurfrakturen beim Polytrauma können primär operativ stabilisiert werden.  2011 | 0 |
| 3.73 | Knieluxationen sollen zum frühestmöglichen Zeitpunkt reponiert werden.  2011 | A |
| 3.75 | Instabile proximale Tibiafrakturen und Tibiakopffrakturen können primär stabilisiert werden.  2011 | 0 |
| 3.76 | Tibiaschaftfrakturen sollten operativ stabilisiert werden.  2011 | B |
| 3.77 | Distale Unterschenkelfrakturen einschließlich artikulärer distaler Tibiafrakturen sollten operativ stabilisiert werden.  2011 | B |
| 3.78 | Sprunggelenksfrakturen sollten primär stabilisiert werden.  2011 | B |
